# Predicting mortality among ischemic stroke patients using pathways-derived polygenic risk scores

**DOI:** 10.1038/s41598-022-16510-x

**Published:** 2022-07-19

**Authors:** Jiang Li, Durgesh Chaudhary, Christoph J. Griessenauer, David J. Carey, Ramin Zand, Vida Abedi

**Affiliations:** 1 Department of Molecular and Functional Genomics, Weis Center for Research, Geisinger Health System, Danville, PA 17822 USA; 2 Neuroscience Institute, Geisinger Health System, Danville, PA 17822 USA; 3 Department of Public Health Sciences, College of Medicine, The Pennsylvania State University, Hershey, PA USA; 4 Research Institute of Neurointervention, Paracelsus Medical University, Salzburg, Austria

**Keywords:** Stroke, Genome-wide association studies, Predictive medicine

## Abstract

We aim to determine whether ischemic stroke(IS)-related PRSs are also associated with and further predict 3-year all-cause mortality. 1756 IS patients with European ancestry were randomly split into training (n = 1226) and testing (n = 530) groups with 3-year post-event observations. Univariate Cox proportional hazards regression model (CoxPH) was used for primary screening of individual prognostic PRSs. Only the significantly associated PRSs and clinical risk factors with the same direction for a causal relationship with IS were used to construct a multivariate CoxPH. Feature selection was conducted by the LASSO method. After feature selection, a prediction model with 11 disease-associated pathway-specific PRSs outperformed the base model, as demonstrated by a higher concordance index (0.751, 95%CI [0.693–0.809] versus 0.729, 95%CI [0.676–0.782]) in the testing sample. A PRS derived from endothelial cell apoptosis showed independent predictability in the multivariate CoxPH (Hazard Ratio = 1.193 [1.027–1.385], *p* = 0.021). These PRSs fine-tuned the model by better stratifying high, intermediate, and low-risk groups. Several pathway-specific PRSs were associated with clinical risk factors in an age-dependent manner and further confirmed some known etiologies of IS and all-cause mortality. In conclusion, Pathway-specific PRSs for IS are associated with all-cause mortality, and the integrated multivariate risk model provides prognostic value in this context.

## Introduction

A recent report from the Global Burden of Diseases, Injuries, and Risk Factors Study (GBD) has shown a substantial increase in the annual number of strokes and secondary deaths^1^. The current prediction models on post-stroke mortality vary by setting, observation window, algorithms, the breadth of clinical variables, and overall usefulness^2^. So far, features selected to predict post-ischemic stroke (post-IS) mortality mainly focus on demographics, social, and clinical factors. Identified genetic risk factors have not been integrated into these prediction models, individually or together, as a composite score in either cohort^2^ or longitudinal studies.

Genome-wide association studies (GWAS) on IS and its etiologic subtypes have been conducted for a decade, and more stroke risk loci have been identified^3^. Polygenic risk scores (PRSs) based on the effect sizes estimated from the meta/mega-analyses of GWAS, led by the MEGASTROKE consortium, have proven informative for IS risk stratification^4^ and augmenting subtyping^5^. The short-term or long-term outcomes have become “The Next Big Thing” in the focus of stroke genetics with a great demand for the development of neuroprotective agents^6^.

Post-IS mortality is considered a complex multifactorial trait with known and unknown etiologies. The risk of stroke-related death and stroke hospitalization in monozygotic compared with dizygotic co-twins is increased with the heritability estimated at 0.32 and 0.17, respectively^7^, suggesting that genetic liability contributes to post-stroke mortality. Studies to incorporate genetic variants into the diagnostic/prognostic algorithms for improving post-stroke care are underway^8^. Domain knowledge-based PRS can be used to integrate genetic variants—at the basis of shared biological pathways—and reduce the hypothesis space due to the convergence of gene functions. Pathway-specific PRSs can stratify diseases into subtypes in the UK Biobank with substantially greater power than genome-wide PRSs^9^. Emerging pathway-specific PRS offers profound insight into the complex disease and treatment response heterogeneity, prioritizes biologically tractable therapeutic targets, and provides an alternative path to precision medicine and outcome prediction in multiple disorders^10–15^.

Through a regularized regression model to integrate multiple sets of GWAS summary statistics on stroke and its modifiable clinical risk factors, a metaGRS has been developed to determine its independent predictability for IS^16^. Our previous study has identified several pathway-specific PRSs that are significantly associated with IS or IS subtypes^5^. This study is aimed to evaluate whether we can prioritize mortality-related PRSs from these candidates through a regularized regression and further demonstrate their independent predictability in an integrated mortality prediction model.

## Method

**Figure 1:**
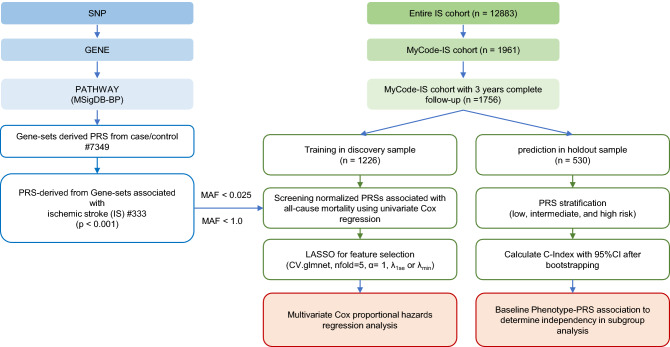
A flow chart for the strategy of the entire analysis. Domain Knowledge-based approach to identify disease-relevant pathway-specific PRSs and further predict all-cause mortality using an integrated multivariate Cox proportional-hazards model with features selected by LASSO.

The Geisinger MyCode Community Health Initiative is a health system-based population representing a geographically defined population who visit Geisinger clinics from East and Central Pennsylvania and is enrolled in the MyCode genotyping and exome sequencing program^17^. We have previously shown that PRS augments stroke subtyping in a retrospective cohort study from September 2003 to May 2019 using data from the MyCode population^5^. A total of 12,883 IS patients were identified, and their data was extracted from the updated Geisinger Neuroscience Ischemic Stroke (GNSIS) database^18^, of which 15.2% (1961) were enrolled in the MyCode program and met the inclusion/exclusion criteria (Fig. 1). A total of 1756 out of 1961 patients included in this study had 3-years of follow-up. We also identified 19,806 MyCode patients with index age ≥ 69 but without the *International Classification of Diseases (ICD)*, *Ninth or Tenth Revision* codes for IS. They were only used to prioritize disease-associated pathway-specific PRSs, as shown in a previous study^5^.

The Geisinger institutional review board approved this study for the use of de-identified data. Informed consent was obtained for all MyCode patients and/or their legal guardians included in this study. All methods were performed in accordance with the relevant guidelines and regulations. Information for genetic variants and their weight^3^ in the construction of each PRS is publicly available at the MEGASTROKE website (https://www.megastroke.org/); Information for candidate gene-sets selected for this study was previously identified^5^.

### The outcome of interest and clinical risk factors

Long-term mortality was defined as the hazard of death due to all causes within 3-year (primary outcome variable) after incident stroke. For comparative assessment, we further evaluated all-cause mortality within 1-year and 5-year after the incident stroke. All-cause mortality is a more robust end-point than a specific cause of death^19^.

We calculated for each patient from date of the index stroke till death or date of the end of follow-up. The data from all encounter types were extracted and processed to ensure the comprehensiveness of the follow-up information. The last encounter of patients was also recorded to ensure that patients were active. Filters were applied to ensure that the relevant variables were captured within the desired time frame while maintaining the order of events. This database was cross-checked with the Social Security Death Index to reflect updated information on the vital status. The 3-year all-cause mortality rate was calculated by dividing the total number of patients who died within three years after the initial stroke event by the total number of IS patients with three-year follow-up (Table 1). Standardized mortality ratios (SMRs) were calculated as the ratio of observed (mortality rate in the MyCode IS) to expected deaths (mortality rate in the entire GNSIS database of Geisinger) in the duration of the follow-up.

**Table 1:** Baseline characteristics in the retrospective study cohort.

Dataset	Training (n = 1226)			Testing (n = 530)			All Dataset (n = 1756)			ANOVA or Chi-square	
Feature	N	Mean ± SD or Frequency(%)	%Missing	N	Mean ± SD or Frequency(%)	%Missing	N	Mean ± SD or Frequency(%)	%Missing	*F* or χ2 statistics	pvalue
Age at Index (≥ 66.8))	1226	66.42 ± 12.03	0	530	66.35 ± 12.76	0	1756	66.4 ± 12.25	0	0.01	0.91
Age at Index(≥ 66.8))	614	50.1	0	265	50	0	879	50.1	0	0	0.98
Hypertension	952	77.7	0	399	75.3	0	1351	76.9	0	1.17	0.28
Systolic BP	949	132.5 ± 22.43	22.59	418	130.31 ± 18.71	21.13	1367	131.83 ± 21.38	22.15	3.05	0.08
Diastolic BP	949	72.07 ± 11.35		418	71.1 ± 10.54		1367	71.78 ± 11.11		2.22	0.14
BMI	1225	29.6 ± 7.23	< 0.01	528	29.27 ± 7.25	< 0.01	1753	29.5 ± 7.23	< 0.01	0.78	0.38
BMI ≥ 25	905	73.8		365	68.9		1270	72.3		4.17	0.04
BMI ≥ 30	507	41.4		204	38.5		711	40.5		1.16	0.28
Sex (Male)	671	54.7	0	281	53	0	952	54.2	0	0.44	0.51
Atrial fibrillation	311	25.37	0	141	26.6	0	452	25.74	0	0.3	0.59
Coronary Artery Disease	414	33.8	0	186	35.1	0	600	34.2	0	0.29	0.59
Diabetes	393	32.1	0	171	32.3	0	564	32.1	0	0.01	0.93
Dyslipidemia	601	49	0	247	46.6	0	848	48.3	0	0.87	0.35
Ever smoke	679	55.4	19.25	289	54.5	18.11	968	55.1	18.91	0.55	0.46
Alcohol	180	14.7	40.46	73	13.8	40.19	253	14.4	40.38	0.32	0.57
NIHSS ≥ 7	163	21.5	38.17	70	21	36.98	233	21.3	37.8	0.04	0.84
NIHSS ≥ 10	94	12.4		47	14.1		141	12.9		0.58	0.45
NIHSS ≥ 16	51	6.7		26	7.8		77	7.1		0.4	0.53
Death within 5 yr	261	21.3	0	123	23.2	0	384	21.9	0	0.8	0.37
Death within 3 yr	193	15.7	0	91	17.2	0	284	16.2	0	0.56	0.46
Death within 1 yr	96	7.8	0	48	9.1	0	144	8.2	0	0.74	0.39

The study cohort was split into training and testing datasets. We also provided clinical characteristics stratified by dichotomized age at index stroke in eTable 2. ANOVA or Chi-square test was selected for quantitative or qualitative measures, respectively. Abbreviations; ANOVA = analysis of variance; BMI = body mass index; BP = blood pressure; Ever Ssmoke: Defined as ever smoke at least one cigarette per day within the three years prior to the event; Alcohol: Defined as consuming more than 200 g of pure alcohol per week. All continuous variables are reported as means with standard deviations, and dichotomous variables are reported as percentages. Patients with a BMI of 18.5 to 24.9 kg/m2 and BMI of 25 to 29.9 kg/m2 were categorized as normal weight and overweight, respectively. Patients with a BMI of 30 kg/m^2^ and above were categorized as obese. NIHSS ≥ 7 was dichotomized NIHSS by score ≥ 7. Similar for NIHSS ≥ 10 and NIHSS ≥ 16.

The diagnosis of clinical risk factors was based on structured data captured in the EHR using ICD9/10 codes^5^.

The demographic and the frequency of clinical risk factors, as shown in Table 1, were comparable to some previously reported cohorts^19–22^. This was a quality control step to avoid significant coding bias for comorbidities.

### Missing clinical data and imputation

For the self-reported variables (such as alcohol, smoking) the missing value was replaced by zero. The BMI and systolic and diastolic blood pressures were imputed by MICE 2lpan, an appropriate strategy as we previously demonstrated^20^. No imputation was conducted for NIHSS (missingness at 37.8%) as there is no consensus strategy to impute this variable. Table 1 lists the missingness levels in all the variables. Variables with a high level of missingness (such as NIHSS) were not included in the final model. However, we explored the potential associations with PRSs in the prediction model to determine independence or possible interaction.

### Genotyping, imputation, and quality control

Samples were genotyped using Infinium OmniExpress Exome array (Illumina) and GSA-24v1-0 array (Illumina). Genotypes for both cohorts were imputed to HRC.r1-1 (Haplotype Reference Consortium reference panel, version r1.1) EUR reference genome (GRCh37 build) separately using Michigan Imputation Server, which employed Eagle v2.3 and Minimac4 as the phasing and imputation algorithm, respectively. Samples with a genotyping rate below 95% were excluded. SNPs with imputation info score < 0.7, minor allele frequency (MAF) < 1%, and significant deviation (*p* < 10^–4^) from Hardy–Weinberg Equilibrium (HWE) were removed. A pruned set of SNPs (608,437) was generated from high quality genotyped SNPs (MAF > 0.05, HWE *p* > 0.0001, LD pruned with *r*^2^ between SNPs < 0.2). A fast PCA (“https://www.cog-genomics.org/plink/2.0/”) using 1 KG phase III (2014 version) as the reference genome indicated that all the selected cases and controls were of EUR.

### PRS construction and estimation

PRS_avg_^5^ was constructed by PRSice-2^21^, with the algorithm, $${PRS}_{j}= \sum_{i}\frac{{X}_{ij}{\widehat{\beta }}_{i}}{{m}_{j}}$$, which was calculated by the number of observed effective allele ($${X}_{ij})$$ for each variant multiplied by the corresponding effect size ($${\widehat{\beta }}_{i})$$ derived from the MEGASTROKE, divided by the number of alleles ($${m}_{j}$$) included in PRS from that individual, and finally sum of all from that individual (*j*). All PRSs were normalized first ($${X}^{^{\prime}}= \frac{X-\mu }{\sigma }$$), where *μ* is the mean and *σ* is the standard deviation of the *X* variable.

Pathway-specific PRSs were created using the gene-set specific PRS algorithm implemented in PRSice-2. We reconstructed domain knowledge-based PRS using gene-sets derived from the Gene Ontology (GO) Biological Process under two MAF thresholds (MAF < 0.025 or < 1.0), which represent low-frequency common variants (SNP_n_ = 68,379) or all variants (SNP_n_ = 231,307) accordingly. LD-clumping using the following PLINK command: –clump-p1 1,–clump-kb 1000, – clump-r2 0.3 was applied to all common variants matched to the variants collected in the base files, the summary statistics from MEGASTROKE . A total of 7349 GO pathways of the biological process and their related genes were defined by Molecular Signatures Database (https://www.gsea-msigdb.org/gsea/msigdb/index.jsp).

The final report of association with IS was a result of meta-analyses of discovery (n = 1184/10,983 for case/control) and replication (n = 951/8823 for case/control) datasets^5^. The sex and five major principal components (PCs) were included as covariates in the logistic regression model. We only considered the signal of association valid given at least 25 SNPs per gene-set. The pathway-specific PRSs associated with IS with *p* < 0.001 were considered disease-relevant PRSs for the subsequent analyses. The best fit pathway-specific PRS per gene-set was selected from either MAF < 0.025 or MAF < 1.0. A total of 333 PRSs (114 from MAF < 0.025 and 219 from MAF < 1.0) were identified as potential candidates for the feature selection. In this study, we used self-contained p value for filtering but not for ranking of pathway-specific PRSs. In order to reduce the need of extensive computational resources, we did not calculate competitive p value using the permutation approach.

### Evaluation of the PRSs prediction model

The PRSs were created at one time for the entire cohort. We randomly split the cohort into 70% training (n = 1226) and 30% testing datasets for model training and testing. eFigure 2 presents a general overview of each normalized PRS output distribution directly from the risk score calculation.

Univariate Cox proportional hazards model was conducted to determine if any PRSs or nongenetic variables affected 3-year mortality results in the training dataset. We set up the threshold of an unadjusted p-value for this association in a stepwise manner to consider different numbers of PRSs during the feature selection and model fine-tuning process. Four tiers of significance of this association were established with *p* < 0.1, *p* < 0.025, *p* < 0.05, and *p* < 0.01. The least absolute shrinkage and selection operator (LASSO) method^22^ in multivariate CoxPH model, an *L1* penalization technique, was applied for feature selection. It forced some regression coefficient estimates to be exactly zero, thus achieving variable selection while shrinking the remaining coefficients toward zero to avoid the overfitting and overestimation caused by data-based model selection.

The partial likelihood for Cox models for β was calculated by^22^.

$$L\left( \beta \right) = \mathop \prod \limits_{r \in D} \frac{{{\text{exp}}\left( {\beta^{T} X^{{j_{r} }} } \right)}}{{\{ \mathop \sum \nolimits_{j \in R} {\text{exp}}(\beta^{T} X^{j} )\} }}$$

where *X* = (${X}_{1}, {X}_{2}, \dots , {X}_{p})$ , a vector of *p* predictors; $\beta =({\beta }_{1}, {\beta }_{2,}$ … , ${\beta }_{p})$ ^*T*^ , estimates of regression coefficients in the proportional-hazards model; *D*, the set of indices of the failures (death); ${R}_{r}$, the set of indices of the individuals at risk at time ${t}_{r}$– 0; ${j}_{r}$ denote the index of the observation failing at time ${t}_{r}$.

The penalized partial likelihood for Cox models was calculated by.

$$L\left( \beta \right) - \mathop \sum \limits_{x = 1}^{p} p_{\alpha } , \lambda \left( {\left| {\beta_{x} } \right|} \right)$$

where α = 1, ${p}_{\lambda }\left(\left|{\beta }_{x}\right|\right)= \lambda |{\beta }_{x}|$, *x ∈ p,* a vector of *p* covariates, and $\sum \left|{\upbeta }_{x}\right| \le s$, where *s* > *0* is a user-specified parameter. $\lambda$ is a penalty coefficient that was selected from a simulated vector.

Briefly, we called our CV.glmnet function to fit with the lasso penalty (alpha = 1), and using CV (nfolds = 5) to select optimal λ. We set the maximum number of iterations to 10,000 because our data is relatively high dimensional, so more iterations were needed for convergence. We extracted both λ, λ_min,_ and λ_1se_, and the λ used in the final model was determined by the c-statistics calculated from the testing sample. This (to select λ_min,_ or λ_1se_) was the step to assess the bias-variance tradeoff. Variables used in the final risk model(s) and their effect sizes are shown in eTable 1. We then refit a multivariate CoxPH model using nongenetic variables or selected PRSs with non-zero regression coefficients estimated from the training dataset by Cross-Validation.glmnet (R package ‘glmnet’).

The partial log-likelihood (*LL*) deviance from fivefold cross-validation (CV),

$$\widehat{{CV}}{\text{i}}\left( \lambda \right) = LL\left( {\hat{\beta }_{{ - {\text{i}}}} \left( \lambda \right)} \right) - LL_{{ - {\text{i}}}} \left( {\hat{\beta }_{{ - {\text{i}}}} \left( \lambda \right)} \right)$$

where $\hat{\beta }_{{ - {\text{i}}}} \left( \lambda \right)$ is the parameter estimate leaving out part *i* of the data, and $LL_{{ - i}}$ is the log-likelihood leaving out part *i* of the data.

Schoenfeld residuals from the Cox models were examined to access possible departures from model assumptions.

### Model assessment, fine-tuning, and comparison

We assessed the performance of the multivariate model in the prediction of 3-year mortality with concordance probability (C-index) which was computed on the 30% testing dataset using methodology described elsewhere^23^, which was recognized as Uno’s C-statistic for right censored data. To determine whether the final multivariate model was working better than random chance, we can empirically compute the null C-index distribution by generating linear predictors from a normal distribution and comparing observed C-index to null distribution using C-statistics implemented in the R survC1 package. This C-index tells how well the given prediction model works in predicting events (mortality) that occur in the time range from 0 to ‘tau’, which was set as 3-year. To quantify the improvement of predictability using an integrated model with additional pathway-specific PRSs superior to the base model (clinical risk factors only), we calculated continuous Net Reclassification Index (NRI), Integrated Discrimination Improvement Index (IDI), and median improvement by R package ‘survIDINRI’^23–25^.

We defined subjects who have events by ${t}_{0}$ as cases (i.e. ${T}^{0}\le {t}_{0}$) and those who are event-free as controls (i.e. ${T}^{0}> {t}_{0}$)., ${\widehat{p}}_{2}{(Z}_{\left(2\right)}^{0};{t}_{0})$ and ${\widehat{p}}_{1}{(Z}_{\left(1\right)}^{0};{t}_{0})$ were defined as two approximations to $\widehat{p}({Z}^{0};{t}_{0})$ by two survival models (e.g. integrated and base models), where ${Z}_{\left(2\right)}^{0}$ and ${Z}_{\left(1\right)}^{0}$ denote the corresponding covariate vectors. $\widehat{D}\left({Z}^{0};{t}_{0}\right)={\widehat{p}}_{2}\left({(Z}_{\left(2\right)}^{0};{t}_{0}\right)- {\widehat{p}}_{1}\left({(Z}_{\left(1\right)}^{0};{t}_{0}\right)$, which denotes the change in estimated risk score^23^. The empirical distribution function of $\widehat{D}$ is represented by a **thick** solid line for ${T}^{0}\le {t}_{0}$ and **thin** solid line for ${T}^{0}> {t}_{0}$. If the integrated model gives a better prediction than the base model, it can be expected that $\widehat{D}$ tends to be positive for a case and negative for a control. The class of measures we consider here is a set of global measures for the ‘distance’ between these two distributions of $\widehat{D}$. The distances between two black dots and between two gray dots are the estimation of IDI when $s=0$ and NRI for median risk-score difference, respectively. All the point estimates with 95%CI for three values were calculated based on 2000 perturbation resampling, and the significance of this improvement was present.

### Survival analyses

We conducted survival analysis and assumed all survival times were independent of each other and censoring occurred solely as right censoring and uninformative. All covariates were measured at or surrounding the index date without changing over time. For univariate modeling, 3-year all-cause mortality was also assessed using the Kaplan–Meier estimator for cumulative incidence function in the training dataset. An optimal cutpoint was obtained by a maximally selected rank statistic greater or equal to b (a percentile) to distinguish low and high-risk groups. We approximated the exact conditional *p* value by simulation using conditional *Monte Carlo* (R package ‘maxstat’). For multivariate modeling, the cumulative incidence of mortality over time in the high-risk, intermediate-risk, and low-risk groups (three terciles) was optimized in the training dataset. It was tested on the testing dataset by R ‘survival’ and ‘survminer’ packages. We also conducted pairwise Log-rank tests from the three strata (high-risk, intermediate-risk, and low-risk) for mortality using the Kaplan–Meier estimator. For all analyses, *p* < 0.05 was considered statistically significant. For all the post-hoc pairwise tests, *p* values were adjusted by the Benjamin-Hochberg procedure.

### Subgroup analyses

We split the entire cohort into younger and older stroke subgroups by 1:1 ratio using the median age at the index stroke date. We also evaluated the effect size of the PRS in the 1-year, 3-year, and 5-year univariate CoxPH model in all samples and subgroups. The relationships between PRSs (predictive variable) and clinical risk factors (dichotomized response variable) were evaluated using logistic regression in all models and subgroups.

## Result

### Clinical characteristics

Table 1 summarizes the demographics and clinical characteristics of 1756 IS patients. Overall, 54.20% were male patients with the median age at index stroke of 66.80 years. The mortality rates were 8.20%, 16.2%, and 21.90% for 1-year, 3-year, and 5-year follow-up periods. Accordingly, the standardized mortality ratios (SMR) was 0.47, 0.58, and 0.59, suggesting most of the death occurred in the 1st year post-IS.

Significantly increased frequency of clinical risk factors such as CAD, hypertension, AFib, Diastolic BP, dyslipidemia, diabetes was observed in the older subgroup (eTable 2). The mortality rates significantly increased from 3.31%, 7.87%, and 11.74% to 13.08%, 24.46%, 31.97% for 1-year, 3-year, and 5-year follow-up, suggesting a relatively more benign outcome for younger patients. Sex was not included for feature selection because of insignificant association in the univariate CoxPH model.

### Construction and evaluation of PRS prediction model

**Figure 2:**
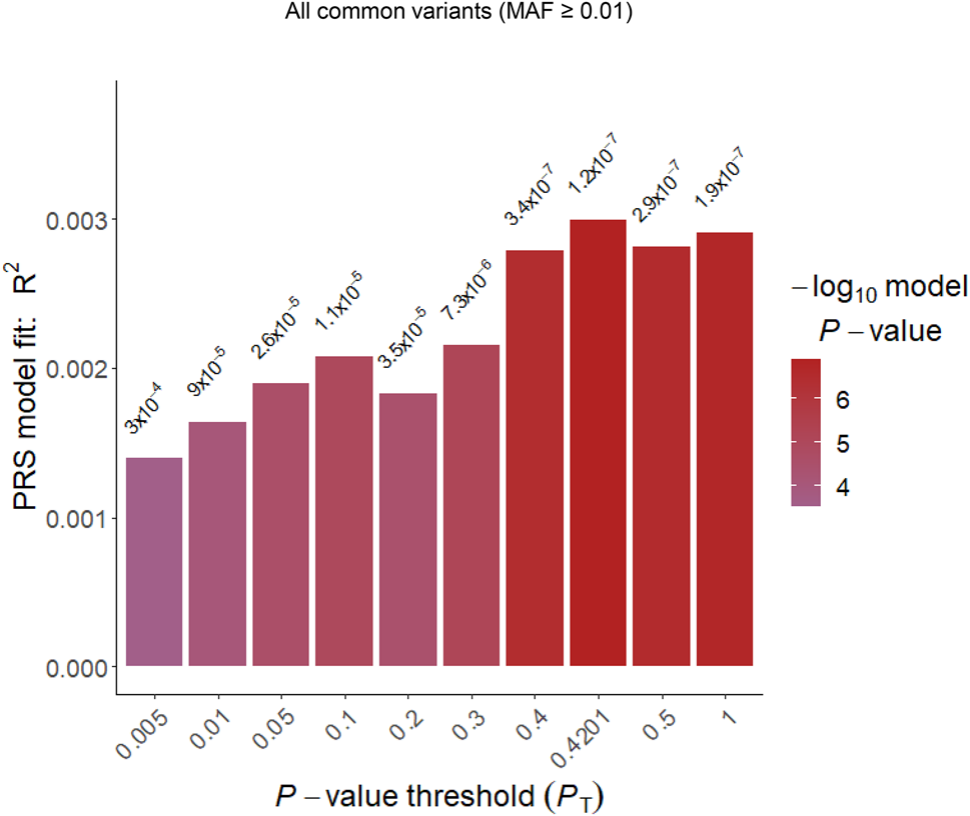
Evaluation of predictive power of PRS derived from all common variants on Geisinger ischemic stroke. PRSs were derived from MEGASTROKE by PRSice-2. LD-clumping using the following PLINK command: –clump-p1 1, –clump-kb 1000, –clump-r2 0.3 was applied to all common variants, resulting in 231,307 SNPs for p-value thresholding in the MyCode IS (n = 1756) versus non-IS (n = 19,806) patients. The results were derived over a range of *p* value thresholds. Nagelkerke pseudo-*R*^*2*^ as shown in the Y-axis, represents how much variation is explained by the model. the X-axis represents the threshold for a base *p* value. *P* value on the top of each bar represents the probability of non-zero regression coefficient with the *F* statistic hypothesis testing of the fit of the intercept-only (PRS excluded) model and PRS included model are equal. The regression model was adjusted by the covariates including sex, and the five main principal components(PCs).

PRS constructed by all common variants for 1756 MyCode IS and 19,806 MyCode patients without IS showed a significant association with IS (p_lowest_ = 1.23 × 10^–7^; Nagelkerke pseudo-*R*^2^ = 0.003). The p-value thresholding primarily confirmed the stability of the observed association (Fig. 2). The events for mortality at 3-year follow-up were 193/1226 (training cohort) and 91/530 (testing cohort). There was no significant difference between training and testing samples except for BMI ≥ 25. PRS constructed by all common variants or all low-frequency variants (MAF < 0.025) showed no association with post-IS mortality in the univariate CoxPH analysis (HR = 1.025, 95%CI [0.882–1.191] or HR = 1.005, 95%CI [0.812–1.243] for 3-year follow-up, respectively).

Common nongenetic risk factors such as age at index stroke, AFib, BMI, CAD, diabetes, dyslipidemia, hypertension, and smoking demonstrated larger HRs than PRSs in the training dataset (eFigure 1).

**Figure 3:**
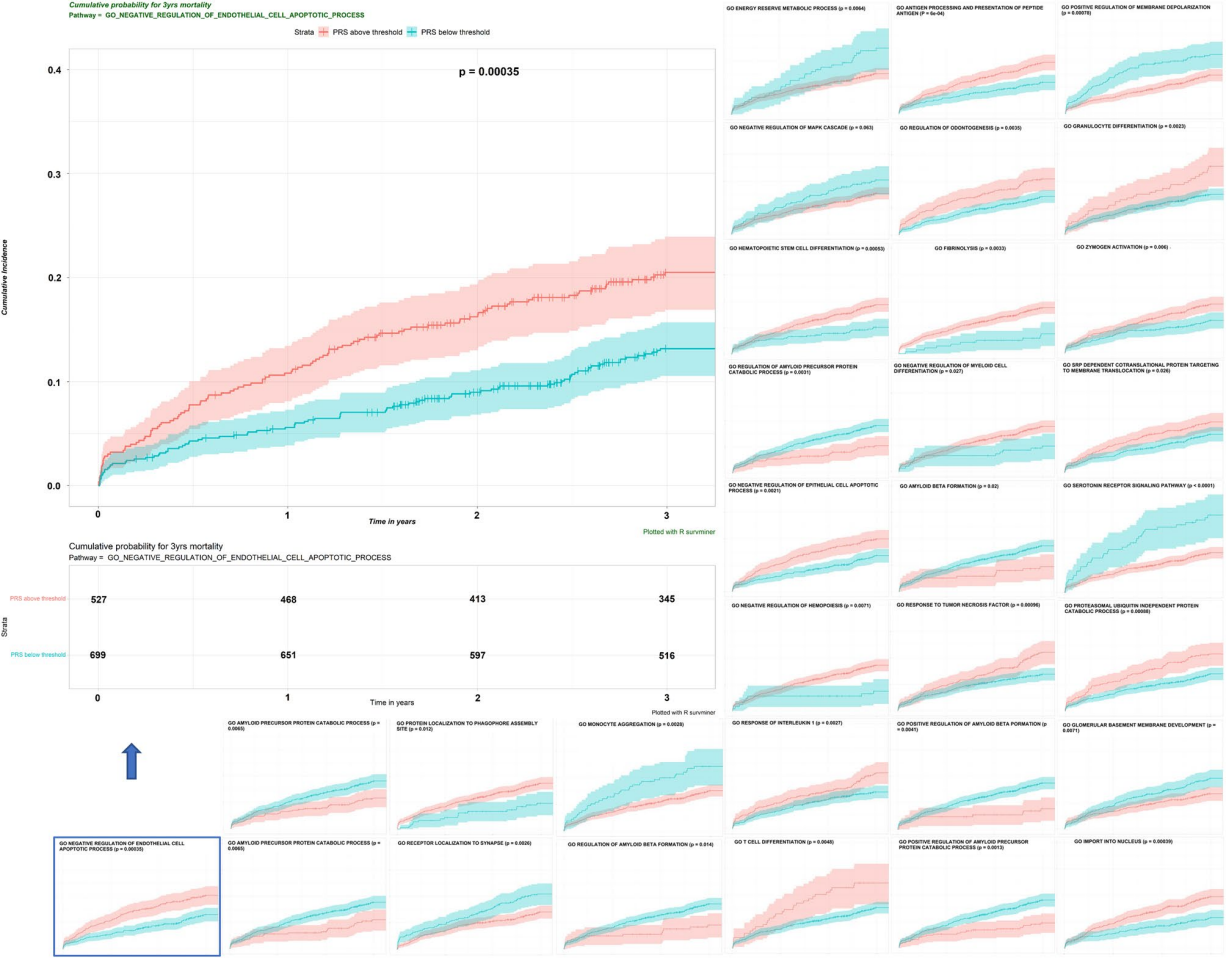
Kaplan–Meier plot of the two groups created by the cutpoint for PRS. All the pathway-specific PRSs for 3-year mortality identified by univariate CoxPH were dichotomized by the corresponding cutpoint, and Kaplan–Meier analysis for each binary PRS was conducted. We simulated the null distribution using the conditional *Monte Carlo* method and compared it with the exact distribution for the log-rank statistic to get the lower bound of the p-value for each pathway-specific PRS. *P* value derived from the Log-rank test was labeled.

After filtering out the paradoxical direction for the regression coefficients of the association between PRS and IS or post-IS mortality, 15 PRSs formed by SNPs with MAF < 1.0 and 16 PRSs formed by SNPs with MAF < 0.025 were treated as disease-relevant pathway-specific PRS, were treated as disease-relevant pathway-specific PRS (p_raw_ < 0.1), eFigure 2 shows the distribution of these PRSs. PRSs constructed from all-common-variants primarily show Gaussian distribution. In contrast, some PRSs derived from low-frequency-variants retained the polynomial distribution patterns, suggesting that low-frequency alleles may only be present in a few patients. These PRSs cannot proportionally stratify the outcome risk. We then obtained the optimal cutpoint for each PRS and dichotomized the patients into high and low-risk groups (eFigure 3). Kaplan–Meier analyses (Fig. 3) showed all 31 PRS candidates significantly distinguished the high and low-risk groups with p_raw_ < 0.05.

The correlation matrix of PRSs (eFigure 4) revealed the hierarchical nature of the GO biological process. For example, some PRSs derived from Amyloid β-related pathways were highly correlated, suggesting their relationship as parent-son terms or sibling terms in the hierarchy of this biological process.

The identified pathway-specific PRSs highlighted the known pathogenesis of IS or post-IS mortality. For example, (1) Amyloid β formation in cerebral small vascular disease; (2) Endothelial apoptosis and inflammation (TNF) in atherosclerosis; (3) Serotonin in platelet aggregation and vascular remodeling; (4) Obesity paradox in post-IS mortality, which was also observed in nonMyCode patients^26^; (5) Coagulation and fibrinolysis in stroke and recurrence.

A total of 8 clinical risk factors and an additional 31, 20, 9, or 3 PRSs with potential prognostic value were identified as selected features after stepwise cutoffs (*p* < 0.1, 0.05, 0.025, 0.01) from the univariate CoxPH (Table 2).

**Table 2:** Predictive performance of the integrated multivariate Cox proportional-hazard regression model with different entry levels for pathway-specific PRSs included for feature selection by LASSO method. The seven clinical risk factors include AFib, BMI, CAD, diabetes, dyslipidemia, hypertension, and smoking.

Univariate *p* value cutoff	Features Input	Features selected (LASSO)	C-Index Mean ± SE [95%CI]	median improvement against base model
< 0.1	31 PRSs + Age	24 PRSs + Age	0.705 ± 0.035 [0.637–0.773]	< 0.001
< 0.05	20 PRSs + Age	16 PRSs + Age	0.684 ± 0.033 [0.619–0.749]	< 0.001
< 0.025	9 PRSs + Age	8 PRSs + Age	0.661 ± 0.036 [0.592–0.731]	0.027
< 0.01	3 PRSs + Age	2 PRSs + Age	0.643 ± 0.034 [0.575–0.710]	0.233
base model	0 PRSs + Age	0 PRSs + Age	0.626 ± 0.024 [0.578–0.674]	NA
< 0.1	31 PRSs + Age + 7	16 PRSs + Age + 7 clinical risk factors	0.754 ± 0.031 [0.693–0.814]	< 0.001
< 0.05	20 PRSs + Age + 7	11 PRSs + Age + 7 clinical risk factors	0.751 ± 0.030 [0.693–0.809]	0.02
< 0.025	9 PRSs + Age + 7	6 PRSs + Age + 7 clinical risk factors	0.740 ± 0.030 [0.680–0.799]	0.066
< 0.01	3 PRSs + Age + 7	2 PRSs + Age + 7 clinical risk factors	0.729 ± 0.028 [0.674–0.783]	0.319
base model	0 PRSs + Age + 7	0 PRSs + Age + 7 clinical risk factors	0.729 ± 0.027 [0.676–0.782]	NA

### Construction of the integrated PRSs prediction model

**Figure 4:**
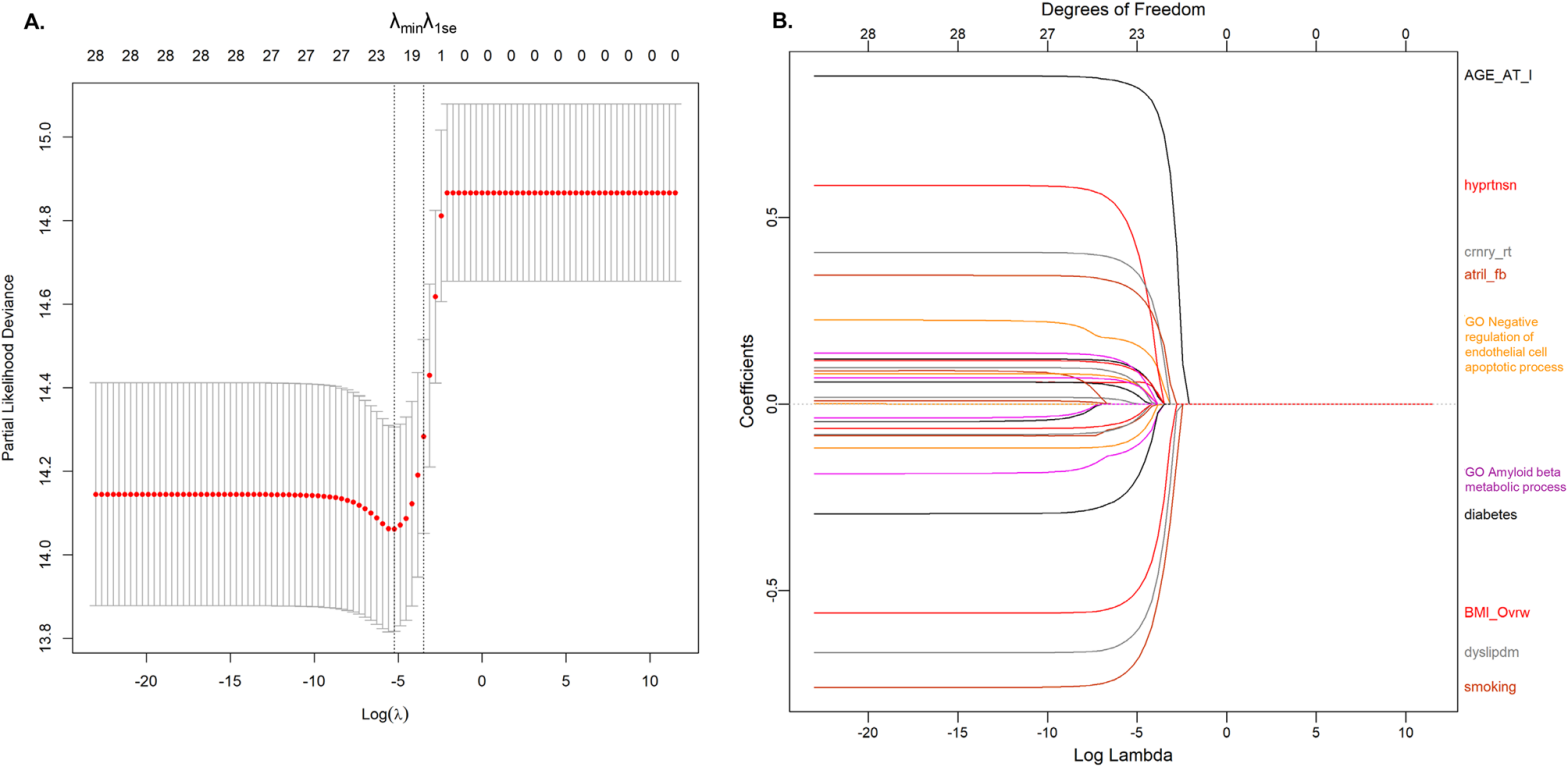
Selection of pathway-specific PRSs using the Least Absolute Shrinkage and Selection Operator (LASSO) Model. Eight clinical and 20 PRS features with *p* < 0.05 from the univariate CoxPH regression were selected for 3-year mortality. Fit the Regularized (LASSO) Cox Model in the training dataset with fivefold cross-validation for the regression coefficients of PRS and nongenetic variables such as age at index stroke. (**A**) X-tile analysis of the features associated with 3-year mortality with *p* value < 0.05, respectively. Y-axis represents partial log likelihood (*LL*) deviance from a fivefold cross-validation, Error bar indicate 95% CIs. The left vertical line in (A) showed where the CV-error curve hits its minimum. The right vertical line in (**A**) shows the most regularized model with CV-error within 1 standard deviation of the minimum. We extract such optimal λ’s. (**B**) Regularization path for the progressively shrinking of the regression coefficients of variables by tuning the λ in the LASSO method with fivefold CV. Variables with bigger absolute regression coefficients were listed. The top ten features with the larger effect size were labeled.

**Figure 5:**
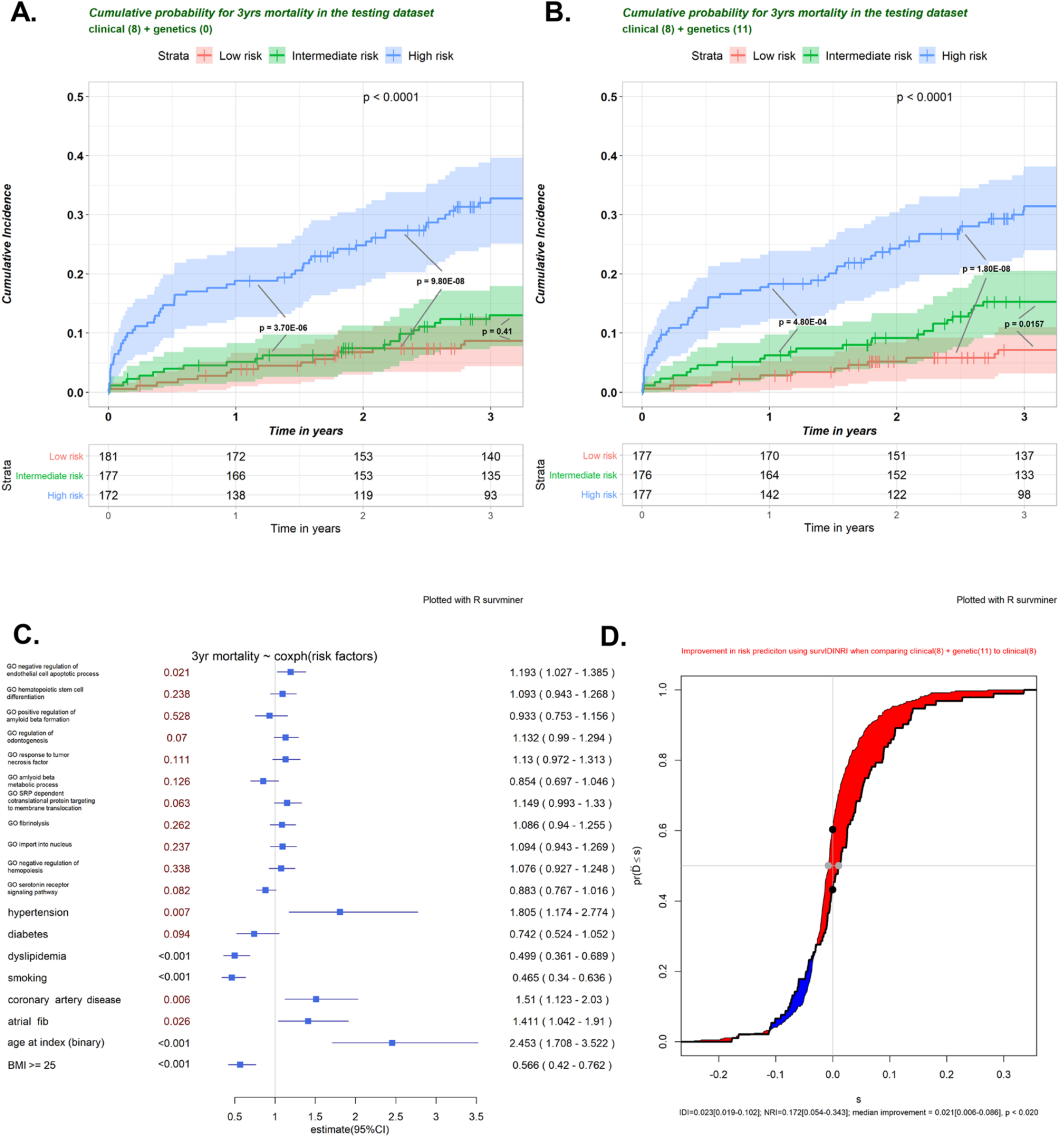
Kaplan–Meier analysis of post-IS cumulative probability for 3 year mortality in the testing sample. Vertical Bar represents right-censored patients. Assuming three risk categories with different survival probability in the testing sample, features included in the multivariate Cox proportional-hazards regression model were selected for calculation of the risk score (${Z}^{T}\widehat{\beta }$) where ${Z}^{T}$ is a vector of covariates and $\widehat{\beta }$ is a vector of estimate of effect size. P-value derived from Log-rank test provided a measure of how well the model stratified risk sets. For the entire analysis, *p* < 0.05 was considered statistically significant. For all the post-hoc pairwise tests, p-values were adjusted by Benjamin-Hochberg procedure. The number of patients at risk was listed in the table. We used candidate features with a *p* value < 0.05 from the univariate Cox regression model as an example for feature selection. We compared Kaplan–Meier curves developed from the base model (**A**) to the integrated model (**B**) with an additional 11 pathway-specific PRSs in the testing sample after the LASSO-based feature selection using the training sample (**C**). The forest plot demonstrated the effect size (HR) for the integrated multivariate Cox regression model in the training sample; (**D**). We calculated continuous Net Reclassification Index (NRI), Integrated Discrimination Improvement Index (IDI), and median improvement by R ‘survIDINRI’ package, as metrics to determine the improvement in prediction when comparing integrated model after additional features selected to the corresponding base model. The additional value of pathway-specific PRS is assessed by the paired difference of risk scores. The empirical distribution function of the paired difference ($\widehat{D}$) between the risk scores (on the probability scale) estimated at ${t}_{0}$ = 3 years using models with and without the inclusion of pathways-specific PRSs. The added value of these PRSs is proportional to the area of the shared region. The vertical difference at s = 0 (between the two black dots, where s scales the region between − 1 and + 1) is NRI (> 0), and the horizontal difference (between the two gray dots) equals the median risk-score difference. Y-axis, cumulative probability; X-axis, s = $\widehat{D}$, the difference between two model risk scores.

The LASSO method was used for feature selection of prognostic PRSs in combination with and without nongenetic features in a multivariate CoxPH (Fig. 4). A total of 16, 11, 5, or 2 PRSs remained to construct the prediction model after filtration by LASSO. Three levels of risk stratification, high-risk, intermediate-risk, and low-risk groups, were proposed. This risk stratification was first conducted in the training dataset (eFigure 5) and then applied to the testing sample (Fig. 5). We also reported the HRs of each variable that remained in the multivariate CoxPH (eTable 1). PRS derived from *GO Negative regulation of endothelial apoptosis* constantly showed a significant association in the final models (i.e., HR = 1.193 [1.027–1.385], p = 0.021 for 3-year mortality), suggesting its robustness as an independent predictor.

### Predictability in the unseen testing sample

A prediction model, based on 11 or 16 disease-associated pathway-specific PRSs, outperformed the base model, as demonstrated by a higher concordance index (0.751, 95%CI [0.693–0.809], 0.754, 95%CI [0.693–0.814], respectively) in the unseen testing sample (Table 2). Estimation of IDI and NRI(> 0) were 0.023 [0.019–0.102] and 0.172 [0.054–0.343] with a significant median improvement (0.021 [0.006–0.086], *p* < 0.020) for the integrated model with 11 pathway-specific PRSs (Fig. 5 and eFigure 6). Compared to the base model, the integrated model (with 11 PRSs) could differentiate not only the high-risk from the intermediate-risk (*p* = 4.80E-4) but also intermediate-risk from the low-risk (*p* = 0.016) (Fig. 5).

In addition, an attempt to dichotomize PRS by a cutpoint showed no benefit at improving the C-index compared to quantitative PRS in the multivariate prediction model.

### Bias assessment and subgroup analysis

**Figure 6:**
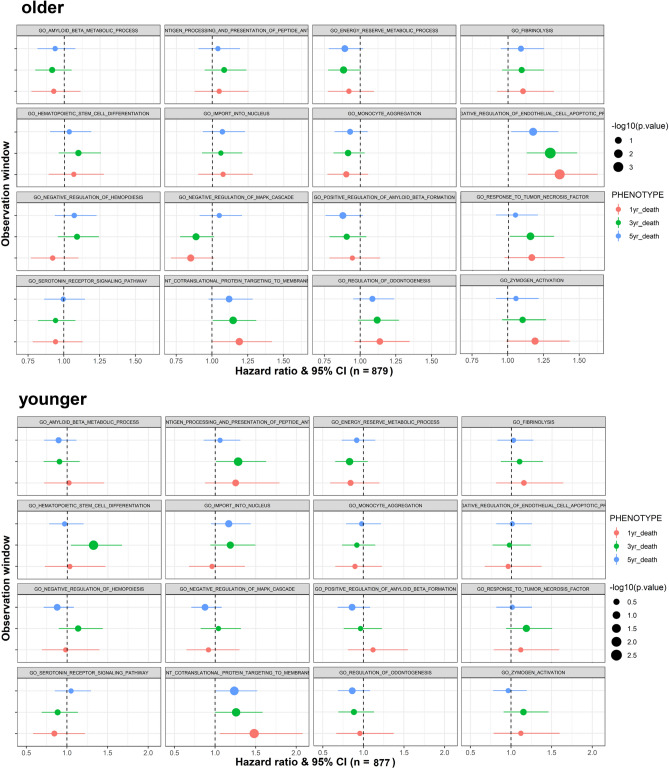
Hazard ratio of each pathway-specific PRS for 1-year, 3-year, and 5-year calendar period of follow-up in all samples as well as subgroups stratified by age at index stroke. The HRs were calculated by the univariate Cox proportional-hazards regression model. The X-bar represents 95%CI of the effect size. Top 16 PRSs were selected from 31 PRS candidates with p-value < 0.1 in association with 3-year mortality from the initial univariate Cox regression model in the training dataset. (**A**) older stroke subgroup; (**B**) younger stroke subgroup.

Since the prognosis of younger stroke is generally considered benign (eTable 2), a more sensitive approach would be to compare the effect size of PRS in association with mortality in a subgroup of age-stratified stroke patients. The forest plots (Fig. 6) revealed the HRs at 1-year, 3-year, and 5-year follow-up for each pathway-specific PRS. Some HRs shifted gradually to the left (e.g., *GO negative regulation of MAPK cascade*) or to the right (e.g., *GO response to Interleukin 1*) over time. In contrast, others showed uncertainty (e.g., *GO positive regulation of membrane depolarization*). Generally, pathway-specific PRSs showed a larger effect size solely in the older subgroup. Secondary analysis of the trends of HR for post-IS mortality showed an increase over time in *GO negative regulation of endothelial cell apoptosis* among older participants, a decline over time in *GO positive regulation of membrane depolarization* among younger participants, and an increase over time in *GO response to Interleukin 1* among older and younger participants (Fig. 6).

*GO negative regulation of endothelial cell apoptosis*; the most predictable PRS demonstrated a significant effect only in the older subgroup suggesting the endothelial injury plays an essential role in post-IS mortality, particularly in the older patients. It was noted that hypertension was the only clinical risk factor showing a trend of association with this PRS with the same direction for both (OR = 1.174, 95%CI [0.975–1.418], *p* = 0.093 for old) (eFigure 7). In a bivariate CoxPH model after adjustment for hypertension, this PRS was still associated with 3-year mortality in all (*p* = 0.044) and the older subgroup (*p* = 0.006), suggesting its independent predictability. After adding an interactive term (PRS: hypertension) into the bivariate CoxPH model, no significant interaction was identified in the older subgroup (*p* = 0.421).

The PRS derived from the *GO serotonin receptor signaling pathway* showed a significant association with IS. A similar trend of association with 3-year mortality was observed in the same direction for the association independent of age at index stroke. This PRS also showed a significant association with CAD regardless of age group (OR = 0.835, 95%CI [0.711–0.978], *p* = 0.027 for younger; OR = 0.869, 95%CI [0.048–0.755], *p* = 0.048 for older).

In the complete case analysis, NIHSS showed significant association with 3-year mortality (HR = 2.978, 95%CI[2.038–4.353] for NIHSS ≥ 16; HR = 2.203, 95%CI[1.597–3.039] for NIHSS ≥ 10). PRS derived from *GO negative regulation of endothelial apoptosis* still show a trend of association after controlling for NIHSS ≥ 16 (*p* = 0.074) and NIHSS ≥ 10 (*p* = 0.082), suggesting this PRS was a partially independent predictor after controlling for NIHSS in the bivariate CoxPH.

## Discussion

This is the first study to predict post-IS all-cause mortality by integrating pathway-specific disease-relevant PRSs into a nongenetic multivariate CoxPH model. In this study, model optimization was performed by applying regularized regression, LASSO, and cross-validation. Despite the smaller effect size (HR < 1.50) for these PRSs compared to nongenetic risk factors identified from the initial univariate CoxPH model, some demonstrated independent predictability in the final multivariate CoxPH model. These included PRS derived from *GO negative endothelial cell apoptotic process regulation and GO Hematopoietic stem cell differentiation*. The integrated model outperformed the clinical-only model significantly. Our results corroborated the capability of PRSs in refining the model’s predictability in the testing dataset. The subgroup analysis results highlighted several pathways associated with IS and post-IS mortality, particularly in the older subgroup. The correlation between PRSs and modifiable clinical risk factors indicated that several pathways might also contribute to modifiable clinical risk factors, suggesting horizontal or vertical pleiotropisms.

The importance of *GO Negative regulation of endothelial cell apoptotic process* in ischemic heart disease has been highlighted in basic science research^27–29^. Understanding the molecular mechanisms involved in the regulation of endothelial cell survival and apoptosis may provide new therapeutic targets to enhance angiogenesis in tissue-ischemia^27^. As the fundamental cause of myocardial infarction and stroke, atherosclerosis involves leukocyte accumulation in the arterial wall and hematopoiesis. Alternatively, hematopoietic stem cell differentiation produces red blood cells, platelets, and leukocytes, contributing to this pathological process^29^. This study provides genetic evidence to support that the abnormality of these pathways due to genetic liability might account for the pathogenesis of the ischemic cerebral vascular disease and post-IS mortality. These results highlight the importance of managing the risk that the identified PRSs stratify in combination with clinical factors.

The control of risk factors is the key to prevention; however, given that these pathway-specific PRSs are nonmodifiable, it is essential to conduct targeted post-stroke surveillance and personalized management on the flagged high- or moderate-risk patients.

Pioneer studies have shown the predictability of PRS in atherosclerotic cardiovascular disease and its outcome^30^. However, the small effect size, limited predictability, or lack of independence in multivariate prediction model^31^ make the predictability of PRS less practical. Nevertheless, incorporating PRSs in the models could still be helpful for populational screening to exclude ‘low-risk individuals. The healthcare resources could be more effectively distributed to care for those needing them the most^32^. Therefore, PRS-false-positive patients classified as “high- or intermediate-risk” would only benefit from the more comprehensive management without harming the high-risk patients.

PRSice-2^21^, an ‘LD clumping, and p-value thresholding’ method, also known as ‘C + T,’ was used for PRS construction. This p-value thresholding can be substituted or combined with MAF thresholding. We found that MAF rather than p-value as the thresholding parameter can significantly enhance the power of the association mainly when using low-frequency variants with effect sizes estimated from the summary statistics of MEGASTROKE GWAS on IS subtypes^5^. The p-value thresholding primarily confirmed the stability of this association between PRS and IS. Low-frequency variants contributed more to the heritability of cardiometabolic traits due to negative selection^33^. Our finding echoed the previously identified GWAS hits of IS enriched with subtype-specific SNPs of low MAF^34^. The previous gene sets analyses using PRS constructed by low-frequency variants highlighted the association of IS with top Gene Ontology terms (vascular endothelial growth factor, amyloid precursor protein, atherosclerosis, and others), known etiologies of IS^5^.

### Validation of exiting etiologies and drug-targeting pathways

The results from the subgroup analysis highlighted several β amyloid peptide (Aβ) related pathways associated with IS and post-IS mortality, particularly in the older subgroup. Sporadic cerebral amyloid angiopathy (CAA) is characterized by progressive deposition of Aβ in the walls of cortical and leptomeningeal small arteries, resulting in vascular occlusion, rupture, and brain parenchymal damage^35^. Aβ has been the culprit for Alzheimer’s disease, hereditary cerebral hemorrhage with amyloidosis^36^, and CAA without symptomatic hemorrhage^35^. Aβ induced toxicity includes generating reactive oxygen species, which trigger a signaling pathway to inflammation and apoptosis^37^. Recent studies showed CAA-linked β-amyloid mutations (E22Q and D23N) promoted cerebral fibrin deposits via increased binding affinity for fibrinogen^38^. All the above findings linked CAA, Aβ, apoptosis, inflammation, and fibrinolysis-related pathways, which were identified together in this study.

The PRSs derived from *GO response to Interleukin 1* (IL1) and *GO response to TNF* shared a moderate level of correlation (0.2 < *r* < 0.4). The PRS from the IL1 pathway was filtered out, but the PRS from the TNF pathway remained in the multivariate CoxPH (eTable 1). IL1 is a therapeutic target for all forms of stroke, and several clinical trials of IL1 receptor antagonists have shown promising results^39^. TNF-α is rapidly upregulated after focal ischemic injury of the brain, and inhibition of TNF-α may represent a novel pharmacological strategy to treat IS^40^. These identified inflammation-related pathways indicated a chronic inflammatory response might contribute to post-IS mortality.

The results from the correlation between PRSs and modifiable clinical risk factors (such as AFib, CAD, etc.) indicated that several pathways might also contribute to modifiable clinical risk factors, suggesting horizontal or vertical pleiotropisms. One of the key findings was the association of multiple pathway-specific PRSs with AFib with the same direction for disease risk and mortality risk, particularly in the older subgroup. This includes pathways related to *fibrinolysis, amyloid precursor protein, response to tumor necrosis factor,* and more*.* After adjustment for AFib, these pathway-specific PRSs no longer showed significance (*p* > 0.1), suggesting they were not independent predictors, further emphasizing the clinical importance of AFib management.

The association between PRS derived from the *GO serotonin receptor signaling pathway* and CAD suggested serotonin might play an important role in ischemic heart disease, ischemic cerebral vascular disease, and the post-event (both conditions) mortality in this cohort. The widely investigated serotonin transporter (SERT) functional polymorphisms have been linked to the risk of incidental IS^41^. The mechanism underlying the detrimental effect of serotonin may involve both neuronal and vascular components. The role of serotonin in thrombogenesis and the development of CAD is well-known^42^. High serotonin level in plasma was significantly associated with CAD and cardiac events, particularly in younger age groups (< 70 years). Serotonin modulates excitatory glutamatergic neurotransmission and induces long-term potentiation, an essential mediator of neuroplasticity that supports sensorimotor learning in the post-stroke perilesional cortex^43^.

### Strengths and limitations

Strengths: (1) Optimizing an integrated prediction model that includes multiple pathway-specific PRSs, which may help to cross the boundary between empirically defined subtypes or comorbidities (because of etiologies consolidated at the pathway level); and (2) Demonstrating the power and utility of models when pathway-derived PRSs are included as features along with known clinical risk factors.

Limitations: (1) Single healthcare system cohort with one ethnic background; (2) Limited sample size (lack of power) for a prediction study in subgroups; and (3) Challenges to the survival analysis using EHR data which are often high-dimensional, censored, have high and not-completely-at-random missingness, and low prevalence for the outcome of interest^20^.

Stroke severity mainly affects survival during the very early phase after stroke; the effect of stroke severity on long-term mortality is limited^44,45^. NIHSS was not included in the final model due to a high level of missingness and lack of consensus on imputing this variable. The PRS derived from *GO Negative regulation of endothelial cell apoptotic process* was a partially independent predictor in the bivariate CoxPH after controlling for NIHSS. These prioritized pathway-specific PRSs may lose their independent predictive power when more valuable (nongenetic or genetic) clinical features are considered in the models.

Finally, PRS should be universally applicable to all patients regardless of ethnicity to ensure health equity in the distribution of healthcare resource^46^. Although this study focused on IS patients of any kind with EUR, the strategy can be adapted to cohorts with mixed ancestry or known TOAST subtypes using PRS constructed by the variants with effect size estimated from similar (mixed) ancestry or subtypes^3,47^.

In conclusion, we provide evidence that pathway-specific PRSs for IS are associated with 3-year all-cause mortality. The integrated multivariate risk model provides a better prognostic value for overall survival after IS. Identified PRSs from disease-relevant pathways echoed several known etiologies for IS as well as post-IS mortality. However, we recognize the effect size of individual pathway-specific PRS was still modest and many of these pathway-specific PRS cannot be considered as independent predictors in the final multivariate model. Validating and expanding the model's utility in external cohorts with mixed ancestry will help determine the generalizability of models when PRSs are part of the feature sets.

## Supplementary Information

Supplementary Information 1.

Supplementary Information 2.

## Data Availability

The summary statistics of our GWAS may be shared with a third party upon execution of the data-sharing agreement for reasonable requests. Information for genetic variants and their weight^3^ in the construction of each PRS is publicly available at the MEGASTROKE website (https://www.megastroke.org/); Information for candidate gene-sets selected for this study was previously identified^5^. The codes developed as part of this study are available at TheDecodeLab/Prediction_of_Post_Stroke_Mortality_by_PRS (github.com).

## Abbreviations

AFib: AFib
APP: Amyloid precursor protein
BMI: Body mass index
CAA: Cerebral amyloid angiopathy
CAD: Coronary artery disease
CES: Cardioembolic stroke
C-index: Concordance index
CI: Confidence interval
CoxPH: Cox proportional-hazards model
C-statistics: Concordance statistics
CV: Cross validation
EHR: Electronic Health Record
eQTL: Expression quantitative trait loci
EUR: European ancestry
GO: Gene Ontology
EUR: European ancestry
GWAS: Genome-wide association study
HR: Hazards ratio
HWE: Hardy–Weinberg Equilibrium
ICD: International Classification of Disease
IS: Ischemic stroke
LAS: Large-artery strokes
LASSO: Least absolute shrinkage and selection operator
LD: Linkage disequilibrium
MAF: Minor allele frequency
ML: Machine learning
NIHSS: National Institutes of Health Stroke Scale
OR: Odds ratio
QTL: Quantitative trait loci
PCA: Principal Component Analysis
PRS: Polygenic risk score
SNP: Single nucleotide polymorphism
SVS: Small vessel stroke
TNF: Tumor necrosis factor
T2D: Type II diabetes
TOAST: Trial of ORG 10,172 in acute stroke treatment
