# Supplementary material for: Predicting mortality among ischemic stroke patients using pathways-derived polygenic risk scores
Source: Sci Rep. 2022 Jul 19;12:12358. doi: 10.1038/s41598-022-16510-x (PMC9296485; doi:10.1038/s41598-022-16510-x)
Supplement: Supplementary file 1 — Supplementary Information 1. [file 41598_2022_16510_MOESM1_ESM.pdf]

eFigures and eTables

eTable 1. Summary of the effect size of each variable in the univariate as well as the multivariate Cox model after feature selection by LASSO method in the training dataset.

A. the model only including one clinical risk factor, Age at onset (Binary); B. the model including 8 clinical risk factors.

eTable 2. Clinical characteristics stratified by dichotomized age at index stroke.

eTable 1a. Summary of the effect size of each variable in the univariate as well as the multivariate Cox model after feature selection by LASSO method in the training dataset (not including clinical risk factors).

Features	Association with IS			p value < 0.1 for univariate cox ph			p value < 0.1 for multivariate cox ph			p value < 0.05 for multivariate cox ph			p value < 0.025 for multiivariate cox ph			p value < 0.001 for multiivariate cox ph			Gene-Set
	MAF	Effect/SE	P.value	HR(MEAN/SE[95%CI])	z statistic	p.value	HR(MEAN/SE[95%CI])	z statistic	pvalue	HR(MEAN/SE[95%CI])	z statistic	pvalue	HR(MEAN/SE[95%CI])	z statistic	pvalue	HR(MEAN/SE[95%CI])	z statistic	pvalue	
AGE_AT_ONSET(Binary)				1.96/0.09[1.66-2.32]	7.83	5E-15	3.49/0.17[2.48-4.91]	7.20	6E-13	3.54/0.17[2.52-4.98]	7.29	3E-13	3.74/0.17[2.67-5.23]	7.65	1.96E-14	3.76/0.17[2.69-5.27]	7.71	1.23E-14	
V3259	1	564.91/139.72	5.270E-05	1.27/0.07[1.1-1.46]	3.31	0.001	1.2/0.08[1.04-1.4]	2.46	0.014	1.2/0.08[1.03-1.39]	2.37	0.018	1.25/0.07[1.08-1.45]	3.03	0.002	1.23/0.07[1.07-1.42]	2.85	4.37E-03	GO_NEGATIVE_REGULATION_OF_ENDOTHELIAL_CELL_APOPTOTIC_PROCESS
V992	1	853.53/220.5	1.085E-04	1.22/0.07[1.06-1.4]	2.75	0.006	1.03/0.09[0.86-1.22]	0.29	0.770	1.04/0.09[0.88-1.23]	0.43	0.669	1.08/0.08[0.92-1.26]	0.98	0.328	1.16/0.07[1.01-1.33]	2.14	0.032	GO_HEMATOPOIETIC_STEM_CELL_DIFFERENTIATION
V1359	1	549.11/156.5	4.503E-04	1.21/0.07[1.05-1.39]	2.59	0.010													GO_NEGATIVE_REGULATION_OF_EPITHELIAL_CELL_APOPTOTIC_PROCESS
V6819	0.03	-88.61/26.72	9.140E-04	0.83/0.08[0.71-0.97]	-2.39	0.017	0.9/0.11[0.73-1.1]	-1.04	0.300	0.89/0.11[0.72-1.09]	-1.12	0.262	0.88/0.1[0.72-1.08]	-1.20	0.229				GO_POSITIVE_REGULATION_OF_AMYLOID_BETA_FORMATION
V2007	0.03	230.35/48.97	2.550E-06	1.17/0.07[1.03-1.34]	2.38	0.017	1.16/0.07[1.02-1.33]	2.22	0.026	1.16/0.07[1.02-1.33]	2.21	0.027	1.16/0.07[1.02-1.33]	2.26	0.024				GO_REGULATION_OF_ODONTOGENESIS
V3447	1	1239.64/375.79	9.712E-04	1.18/0.07[1.03-1.36]	2.34	0.019	1.11/0.08[0.95-1.3]	1.35	0.177	1.12/0.07[0.97-1.3]	1.51	0.131	1.14/0.07[0.98-1.32]	1.75	0.081				GO_RESPONSE_TO_TUMOR_NECROSIS_FACTOR
V5675	0.03	-254.72/68.62	2.056E-04	0.84/0.07[0.73-0.97]	-2.33	0.020	0.92/0.11[0.74-1.13]	-0.81	0.416	0.91/0.11[0.74-1.12]	-0.91	0.361	0.91/0.1[0.74-1.11]	-0.97	0.334				GO_AMYLOID_BETA_METABOLIC_PROCESS
V4403	1	506.84/93.02	5.070E-08	1.18/0.07[1.02-1.36]	2.26	0.024	1.06/0.09[0.9-1.26]	0.69	0.488	1.09/0.08[0.92-1.28]	0.98	0.329	1.06/0.08[0.9-1.24]	0.66	0.512				GO_PROTEASOMAL_UBIQUITIN_INDEPENDENT_PROTEIN_CATABOLIC_PROCESS
V821	0.03	-159.96/43.79	2.589E-04	0.84/0.08[0.73-0.98]	-2.24	0.025	0.88/0.08[0.75-1.03]	-1.56	0.118	0.88/0.08[0.75-1.03]	-1.59	0.112	0.87/0.08[0.74-1.02]	-1.69	0.092				GO_POSITIVE_REGULATION_OF_MEMBRANE_DEPOLARIZATION
V6519	1	-1014.54/282.46	3.284E-04	0.85/0.07[0.74-0.98]	-2.20	0.028	0.89/0.07[0.77-1.02]	-1.64	0.101	0.88/0.07[0.76-1.01]	-1.79	0.074							GO_RECEPTOR_LOCALIZATION_TO_SYNAPSE
V2450	1	564.07/157.84	3.521E-04	1.17/0.07[1.02-1.35]	2.19	0.029	1.05/0.08[0.9-1.23]	0.66	0.509	1.06/0.08[0.91-1.24]	0.72	0.470							GO_GRANULOCYTE_DIFFERENTIATION
V1216	0.03	-310.83/61.14	3.710E-07	0.85/0.08[0.73-0.99]	-2.15	0.031													GO_REGULATION_OF_AMYLOID_PRECURSOR_PROTEIN_CATABOLIC_PROCESS
V4219	0.03	62.89/17.01	2.178E-04	1.16/0.07[1.01-1.34]	2.14	0.032	1.14/0.07[0.99-1.32]	1.81	0.070	1.13/0.07[0.98-1.31]	1.72	0.085							GO_SRP_DEPENDENT_COTRANSLATIONAL_PROTEIN_TARGETING_TO_MEMBRANE_TRANSLOCATION
V3699	1	585.81/148.78	8.240E-05	1.17/0.07[1.01-1.34]	2.11	0.034	1.07/0.08[0.92-1.26]	0.88	0.380	1.08/0.08[0.92-1.27]	0.98	0.329							GO_ZYMOGEN_ACTIVATION
V3046	1	311.34/89.28	4.882E-04	1.16/0.07[1.01-1.33]	2.10	0.035	1.05/0.08[0.89-1.23]	0.55	0.583	1.07/0.08[0.91-1.25]	0.83	0.405							GO_FIBRINOLYSIS
V5350	0.03	-262.97/67.18	9.060E-05	0.86/0.07[0.74-0.99]	-2.10	0.035													GO_AMYLOID_PRECURSOR_PROTEIN_CATABOLIC_PROCESS
V5736	1	1343.5/284.17	2.270E-06	1.16/0.07[1.01-1.33]	2.08	0.037	1.09/0.07[0.94-1.26]	1.15	0.251	1.1/0.07[0.95-1.27]	1.27	0.205							GO_IMPORT_INTO_NUCLEUS
V6899	0.03	-140.19/38.54	2.752E-04	0.86/0.07[0.74-0.99]	-2.08	0.038													GO_POSITIVE_REGULATION_OF_AMYLOID_PRECURSOR_PROTEIN_CATABOLIC_PROCESS
V1637	1	1212.42/272.06	8.330E-06	1.16/0.07[1.01-1.33]	2.07	0.038	1.03/0.09[0.87-1.22]	0.35	0.729	1.06/0.08[0.9-1.24]	0.70	0.483							GO_NEGATIVE_REGULATION_OF_HEMOPOIESIS
V4297	1	-442.9/129.72	6.396E-04	0.86/0.08[0.74-1]	-2.00	0.046	0.89/0.07[0.77-1.03]	-1.55	0.120	0.89/0.07[0.77-1.03]	-1.55	0.122							GO_SEROTONIN_RECEPTOR_SIGNALING_PATHWAY
V785	1	977.84/285.7	6.201E-04	1.15/0.07[1-1.33]	1.91	0.056	1.06/0.08[0.91-1.24]	0.77	0.443										GO_ANTIGEN_PROCESSING_AND_PRESENTATION_OF_PEPTIDE_ANTIGEN
V6166	0.03	-62.3/15.06	3.510E-05	0.89/0.06[0.79-1.01]	-1.87	0.062	0.92/0.06[0.81-1.04]	-1.38	0.167										GO_MONOCYTE_AGGREGATION
V6818	0.03	-263.63/51.42	2.940E-07	0.87/0.08[0.75-1.01]	-1.84	0.066													GO_REGULATION_OF_AMYLOID_BETA_FORMATION
V333	0.03	-309/86.99	3.820E-04	0.88/0.07[0.77-1.01]	-1.80	0.072	0.89/0.07[0.77-1.03]	-1.60	0.109										GO_ENERGY_RESERVE_METABOLIC_PROCESS
V3197	1	770.44/205.09	1.723E-04	1.13/0.07[0.99-1.31]	1.75	0.080													GO_NEGATIVE_REGULATION_OF_MYELOID_CELL_DIFFERENTIATION
V1910	1	1098.97/318.28	5.546E-04	1.13/0.07[0.98-1.31]	1.73	0.084	1.02/0.08[0.86-1.2]	0.23	0.821										GO_RESPONSE_TO_INTERLEUKIN_1
V5065	0.03	103.88/28.71	2.966E-04	1.12/0.07[0.98-1.29]	1.72	0.086	1.06/0.07[0.93-1.21]	0.83	0.405										GO_PROTEIN_LOCALIZATION_TO_PHAGOPHORE_ASSEMBLY_SITE
V5029	0.03	-204.41/53.4	1.292E-04	0.88/0.07[0.76-1.02]	-1.70	0.090													GO_AMYLOID_BETA_FORMATION
V3511	0.03	458.52/137.94	8.870E-04	1.13/0.07[0.98-1.3]	1.69	0.090	1.06/0.08[0.9-1.25]	0.72	0.473										GO_T_CELL_DIFFERENTIATION
V939	0.03	-401.55/120.46	8.575E-04	0.89/0.07[0.77-1.02]	-1.67	0.095	0.94/0.07[0.81-1.09]	-0.85	0.396										GO_NEGATIVE_REGULATION_OF_MAPK_CASCADE
V4929	0.03	-218.54/53.55	4.490E-05	0.88/0.07[0.76-1.02]	-1.66	0.096	0.9/0.08[0.77-1.04]	-1.44	0.150										GO_GLOMERULAR_BASEMENT_MEMBRANE_DEVELOPMENT

eTable 1b. Summary of the effect size of each variable in the univariate as well as the multivariate Cox model after feature selection by LASSO method in the training dataset (including clinical risk factors).

Features	Association with IS			p value < 0.1 for univariate cox ph			p value < 0.1 for multivariate cox ph			p value < 0.05 for multivariate cox ph			p value < 0.025 for multiivariate cox ph			p value < 0.01 for multiivariate cox ph			Gene-Set
	MAF	Effect/SE	P.value	HR(MEAN/SE[95%CI])	z statistic	p.value	HR(MEAN/SE[95%CI])	z statistic	pvalue	HR(MEAN/SE[95%CI])	z statistic	pvalue	HR(MEAN/SE[95%CI])	z statistic	pvalue	HR(MEAN/SE[95%CI])	z statistic	pvalue	
hypertension				1.905/0.171[1.364-2.6	3.78	1.58E-04	1.816/0.22[1.18-2.795]	2.714	6.647E-03	1.805/0.219[1.175-2.774]	2.694	7.070E-03	1.858/0.219[1.209-2.856]	2.825	4.730E-03	1.66/0.215[1.089-2.532]	2.354	1.858E-02	
diabetes				0.764/0.133[0.588-0.9	-2.02	4.37E-02	0.742/0.178[0.524-1.052]	-1.676	9.375E-02	0.742/0.178[0.524-1.052]	-1.677	9.359E-02	0.749/0.178[0.529-1.061]	-1.629	1.033E-01	0.729/0.177[0.516-1.032]	-1.784	7.447E-02	
dyslipidemia				0.478/0.127[0.372-0.6	-5.81	6.42E-09	0.507/0.166[0.367-0.702]	-4.092	4.270E-05	0.499/0.165[0.361-0.689]	-4.22	2.440E-05	0.49/0.164[0.355-0.675]	-4.352	1.350E-05	0.48/0.164[0.348-0.661]	-4.49	7.130E-06	
smoking				2.106/0.231[1.339-3.3	3.22	1.27E-03	0.464/0.16[0.339-0.634]	-4.809	1.520E-06	0.465/0.16[0.34-0.636]	-4.795	1.630E-06	0.472/0.159[0.346-0.644]	-4.739	2.150E-06	0.479/0.159[0.351-0.654]	-4.636	3.560E-06	
coronary_artery				1.988/0.119[1.575-2.5	5.79	7.21E-09	1.513/0.152[1.124-2.037]	2.727	6.383E-03	1.51/0.151[1.123-2.03]	2.729	6.343E-03	1.541/0.15[1.147-2.069]	2.874	4.055E-03	1.63/0.149[1.216-2.184]	3.272	1.070E-03	
atrial_fib				2.191/0.121[1.729-2.7	6.49	8.35E-11	1.397/0.155[1.031-1.894]	2.155	3.120E-02	1.411/0.155[1.042-1.91]	2.227	2.597E-02	1.386/0.154[1.024-1.875]	2.112	3.468E-02	1.371/0.154[1.013-1.854]	2.043	4.107E-02	
AGE_AT_INDEX_BINARY				3.506/0.138[2.673-4.5	9.06	1.30E-19	2.455/0.184[1.71-3.524]	4.868	1.130E-06	2.453/0.185[1.708-3.522]	4.862	1.160E-06	2.489/0.184[1.736-3.57]	4.956	7.210E-07	2.479/0.184[1.729-3.555]	4.937	7.940E-07	
BMI_Overweight				0.497/0.12[0.392-0.62	-5.82	5.86E-09	0.577/0.153[0.428-0.779]	-3.592	3.280E-04	0.566/0.152[0.42-0.762]	-3.749	1.780E-04	0.564/0.152[0.419-0.759]	-3.778	1.580E-04	0.552/0.15[0.411-0.742]	-3.949	7.840E-05	
V3259	1	564.91/139.72	5.270E-05	1.27/0.07[1.1-1.46]	3.31	0.001	1.203/0.077[1.035-1.398]	2.415	0.015741	1.193/0.076[1.028-1.385]	2.315	0.021	1.241/0.074[1.073-1.435]	2.911	3.598E-03	1.243/0.074[1.075-1.437]	2.935	3.330E-03	GO_NEGATIVE_REGULATION_OF_ENDOTHELIAL_CELL_APOPTOTIC_PROCESS
V992	1	853.53/220.5	1.085E-04	1.22/0.07[1.06-1.4]	2.75	0.006	1.08/0.076[0.93-1.254]	1.009	0.313206	1.093/0.076[0.943-1.268]	1.181	0.238	1.11/0.073[0.962-1.28]	1.428	0.153	1.152/0.071[1.003-1.323]	2.005	4.497E-02	GO_HEMATOPOIETIC_STEM_CELL_DIFFERENTIATION
V1359	1	549.11/156.5	4.503E-04	1.21/0.07[1.05-1.39]	2.59	0.010													GO_NEGATIVE_REGULATION_OF_EPITHELIAL_CELL_APOPTOTIC_PROCESS
V6819	0.025	-88.61/26.72	9.140E-04	0.83/0.08[0.71-0.97]	-2.39	0.017	0.935/0.109[0.755-1.157]	-0.621	0.53463	0.933/0.109[0.753-1.156]	-0.631	0.528	0.912/0.108[0.738-1.127]	-0.854	0.393				GO_POSITIVE_REGULATION_OF_AMYLOID_BETA_FORMATION
V2007	0.025	230.35/48.97	2.550E-06	1.17/0.07[1.03-1.34]	2.38	0.017	1.133/0.069[0.99-1.296]	1.817	0.069285	1.132/0.068[0.99-1.294]	1.812	0.070	1.133/0.067[0.993-1.294]	1.856	0.063				GO_REGULATION_OF_ODONTOGENESIS
V3447	1	1239.64/375.79	9.712E-04	1.18/0.07[1.03-1.36]	2.34	0.019	1.134/0.079[0.971-1.324]	1.59	0.111753	1.13/0.077[0.972-1.313]	1.594	0.111	1.154/0.076[0.994-1.34]	1.877	0.061				GO_RESPONSE_TO_TUMOR_NECROSIS_FACTOR
V5675	0.025	-254.72/68.62	2.056E-04	0.84/0.07[0.73-0.97]	-2.33	0.020	0.875/0.104[0.714-1.073]	-1.286	0.198583	0.854/0.104[0.697-1.046]	-1.529	0.126	0.875/0.102[0.717-1.068]	-1.314	0.189				GO_AMYLOID_BETA_METABOLIC_PROCESS
V4403	1	506.84/93.02	5.070E-08	1.18/0.07[1.02-1.36]	2.26	0.024													GO_PROTEASOMAL_UBIQUITIN_INDEPENDENT_PROTEIN_CATABOLIC_PROCESS
V821	0.025	-159.96/43.79	2.589E-04	0.84/0.08[0.73-0.98]	-2.24	0.025													GO_POSITIVE_REGULATION_OF_MEMBRANE_DEPOLARIZATION
V6519	1	-1014.54/282.46	3.284E-04	0.85/0.07[0.74-0.98]	-2.20	0.028													GO_RECEPTOR_LOCALIZATION_TO_SYNAPSE
V2450	1	564.07/157.84	3.521E-04	1.17/0.07[1.02-1.35]	2.19	0.029													GO_GRANULOCYTE_DIFFERENTIATION
V1216	0.025	-310.83/61.14	3.710E-07	0.85/0.08[0.73-0.99]	-2.15	0.031													GO_REGULATION_OF_AMYLOID_PRECURSOR_PROTEIN_CATABOLIC_PROCESS
V4219	0.025	62.89/17.01	2.178E-04	1.16/0.07[1.01-1.34]	2.14	0.032	1.169/0.075[1.009-1.355]	2.083	0.037252	1.149/0.075[0.993-1.33]	1.86	0.063							GO_SRP_DEPENDENT_COTRANSLATIONAL_PROTEIN_TARGETING_TO_MEMBRANE_TRANSLOCATION
V3699	1	585.81/148.78	8.240E-05	1.17/0.07[1.01-1.34]	2.11	0.034	1.094/0.083[0.929-1.287]	1.078	0.280943										GO_ZYMOGEN_ACTIVATION
V3046	1	311.34/89.28	4.882E-04	1.16/0.07[1.01-1.33]	2.10	0.035	1.019/0.082[0.868-1.198]	0.232	0.816317	1.086/0.074[0.94-1.255]	1.123	0.262							GO_FIBRINOLYSIS
V5350	0.025	-262.97/67.18	9.060E-05	0.86/0.07[0.74-0.99]	-2.10	0.035													GO_AMYLOID_PRECURSOR_PROTEIN_CATABOLIC_PROCESS
V5736	1	1343.5/284.17	2.270E-06	1.16/0.07[1.01-1.33]	2.08	0.037	1.09/0.077[0.937-1.268]	1.119	0.263	1.094/0.076[0.943-1.269]	1.183	0.237							GO_IMPORT_INTO_NUCLEUS
V6899	0.025	-140.19/38.54	2.752E-04	0.86/0.07[0.74-0.99]	-2.08	0.038													GO_POSITIVE_REGULATION_OF_AMYLOID_PRECURSOR_PROTEIN_CATABOLIC_PROCESS
V1637	1	1212.42/272.06	8.330E-06	1.16/0.07[1.01-1.33]	2.07	0.038	1.074/0.076[0.925-1.248]	0.938	0.348481	1.076/0.076[0.927-1.248]	0.958	0.338							GO_NEGATIVE_REGULATION_OF_HEMOPOIESIS
V4297	1	-442.9/129.72	6.396E-04	0.86/0.08[0.74-1]	-2.00	0.046	0.886/0.072[0.77-1.02]	-1.682	0.092622	0.883/0.072[0.767-1.016]	-1.738	0.082							GO_SEROTONIN_RECEPTOR_SIGNALING_PATHWAY
V785	1	977.84/285.7	6.201E-04	1.15/0.07[1-1.33]	1.91	0.056	1.05/0.075[0.907-1.215]	0.652	0.514326										GO_ANTIGEN_PROCESSING_AND_PRESENTATION_OF_PEPTIDE_ANTIGEN
V6166	0.025	-62.3/15.06	3.510E-05	0.89/0.06[0.79-1.01]	-1.87	0.062	0.919/0.067[0.805-1.048]	-1.258	0.208239										GO_MONOCYTE_AGGREGATION
V6818	0.025	-263.63/51.42	2.940E-07	0.87/0.08[0.75-1.01]	-1.84	0.066													GO_REGULATION_OF_AMYLOID_BETA_FORMATION
V333	0.025	-309/86.99	3.820E-04	0.88/0.07[0.77-1.01]	-1.80	0.072	0.908/0.073[0.786-1.048]	-1.32	0.187										GO_ENERGY_RESERVE_METABOLIC_PROCESS
V3197	1	770.44/205.09	1.723E-04	1.13/0.07[0.99-1.31]	1.75	0.080													GO_NEGATIVE_REGULATION_OF_MYELOID_CELL_DIFFERENTIATION
V1910	1	1098.97/318.28	5.546E-04	1.13/0.07[0.98-1.31]	1.73	0.084													GO_RESPONSE_TO_INTERLEUKIN_1
V5065	0.025	103.88/28.71	2.966E-04	1.12/0.07[0.98-1.29]	1.72	0.086													GO_PROTEIN_LOCALIZATION_TO_PHAGOPHORE_ASSEMBLY_SITE
V5029	0.025	-204.41/53.4	1.292E-04	0.88/0.07[0.76-1.02]	-1.70	0.090													GO_AMYLOID_BETA_FORMATION
V3511	0.025	458.52/137.94	8.870E-04	1.13/0.07[0.98-1.3]	1.69	0.090													GO_T_CELL_DIFFERENTIATION
V939	0.025	-401.55/120.46	8.575E-04	0.89/0.07[0.77-1.02]	-1.67	0.095	0.924/0.074[0.799-1.069]	-1.058	0.289924										GO_NEGATIVE_REGULATION_OF_MAPK_CASCADE
V4929	0.025	-218.54/53.55	4.490E-05	0.88/0.07[0.76-1.02]	-1.66	0.096													GO_GLOMERULAR_BASEMENT_MEMBRANE_DEVELOPMENT

eTable 2. Clinical characteristics stratified by dichotomized age at index stroke.

Dataset	Young Stroke (n=877)			Old Stroke (n=879)			ANOVA or Chi-square	
Feature	N	Mean±SD or Frequency(%)	%Missing	N	Mean±SD or Frequency(%)	%Missing	*F* or χ^2 statistics	pvalue
AGE_AT_INDEX	877	56.83±8.44	0.00	879	75.94±6.82	0.00	2720.50	0.00
AGE_AT_INDEX (≥66.8))	877	50.00	0.00	879	50.00	0.00	NA	NA
Hypertension	606	69.10	0.00	745	84.76	0.00	60.64	6.84E-15
Systolic blood pressure	734	131.57±21.42	16.31	633	132.12±21.34	27.99	0.23	0.63
Diastolic blood pressure	734	73.43±11.40	22.59	633	69.86±10.45	21.13	36.00	2.52E-09
BMI	875	30.89±7.66	0.23	878	28.12±6.49	0.11	18.62	1.79E-05
BMI ≥ 25	698	79.77	0.23	572	65.15	0.11	46.95	7.29E-12
BMI ≥ 30	416	47.54	0.23	295	33.60	0.11	35.34	2.76E-09
Sex (Male)	485	55.30	0.00	467	53.13	0.00	0.84	0.36
Atrial fibrillation	122	13.91	0.00	330	37.54	0.00	128.26	9.86E-30
Coronary Artery Disease	236	26.91	0.00	364	41.41	0.00	41.04	1.49E-10
Diabetes	299	34.09	0.00	265	30.15	0.00	3.14	0.08
Dyslipidemia	432	49.26	0.00	416	47.33	0.00	0.66	0.42
Ever_smoke	544	72.73	14.71	424	62.72	23.09	16.33	5.32E-05
Alcohol	153	29.03	39.91	100	19.23	40.84	13.72	2.12E-04
NIHSS_7above	87	19.3	48.69	146	22.7	26.96	1.83	1.76E-01
NIHSS_10above	46	10.2	48.69	95	14.8	26.96	4.93	2.60E-02
NIHSS_16above	19	4.2	48.69	58	9	26.96	9.35	2.00E-03
Death up to 5yr	103	11.74	0.00	281	31.97	0.00	105.09	1.17E-24
Death up to 3yr	69	7.87	0.00	215	24.46	0.00	89.14	3.68E-21
Death up to 1yr	29	3.31	0.00	115	13.08	0.00	55.74	8.29E-14

eFigure 1. The forest plot showing the hazard ratio with 95%CI (bar) of each nongenetic variables for 3-year mortality in training dataset.

A univariate Cox proportional-hazards regression model was applied to each variable. Patients from the training dataset with 3-year (n = 1226) follow-up were included in this analysis.

eFigure 2. The kernel density plot showing the distribution of each pathway-specific PRS.

31 PRS candidates with p value < 0.1 in association with 3-year mortality from univariate Cox regression model in the training dataset.

eFigure 3. The standardized statistic of log-rank scores as a function of the hypothetical cutpoints of 31 candidate PRS identified by the univariate CoxPH model for the association with 3-year mortality.

The vertical dash line represents the cutpoint of PRS for the maximum of standardized log-rank statistic.

eFigure 4. Correlation matrix for the pathway-specific PRS, sex, and dichotomized age at index stroke.

eFigure 5. Kaplan-Meier analysis of post-IS cumulative probability for 3-year mortality in training and testing datasets.

Assuming 3 subgroups with different survival probability in the training dataset to determine the effect size of each feature included in the multivariate Cox proportional-hazards regression model. P.value derived from Log-rank test was labelled.

eFigure 6. The comparison of integrated and base models to determine the improvement of predictive models including additional features selected from pathway-specific PRSs.

A-D represents the two model comparisons between base and integrated models with additional 16, 11, 6, or 2 pathway-specific PRSs included, respectively. We calculated continuous NRI, IDI, and median improvement, as metrics to determine the improvement in prediction when comparing integrated model after additional features selected to the corresponding base model.

eFigure 7. Logistic regression to determine the association of each pathway-specific PRS with some clinical risk factors for ischemic stroke in all sample as well as subgroups stratified by the dichotomized age at index stroke.

A. older stroke subgroup; B. younger stroke subgroup.

eFigure 1. The forest plot showing the hazard ratio with 95%CI (bar) of each nongenetic variables for 3-year mortality in training dataset.
A univariate Cox proportional-hazards regression model was applied to each variable. Patients from the training dataset with 3-year (n = 1226) follow-up were included in this analysis.

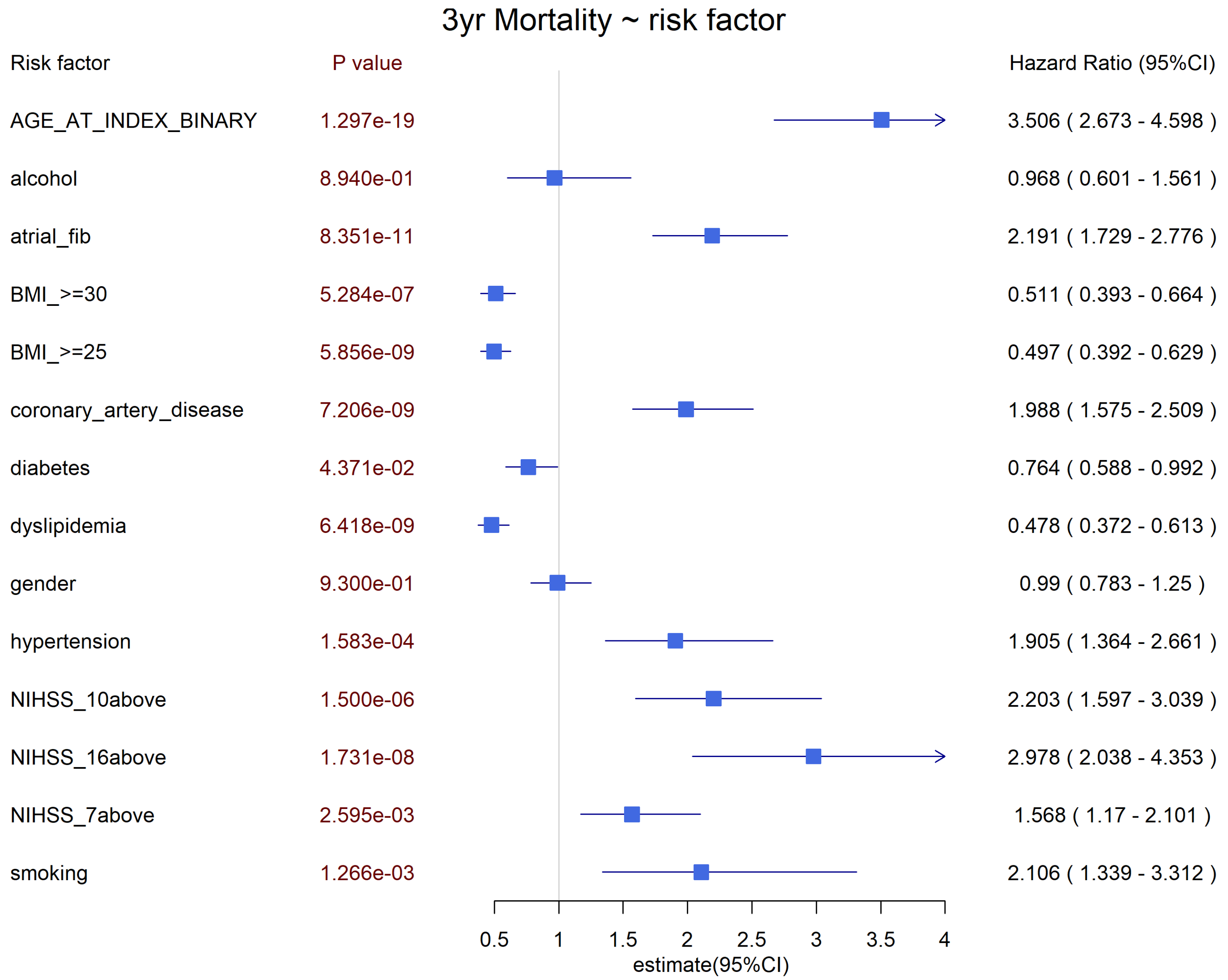

A.

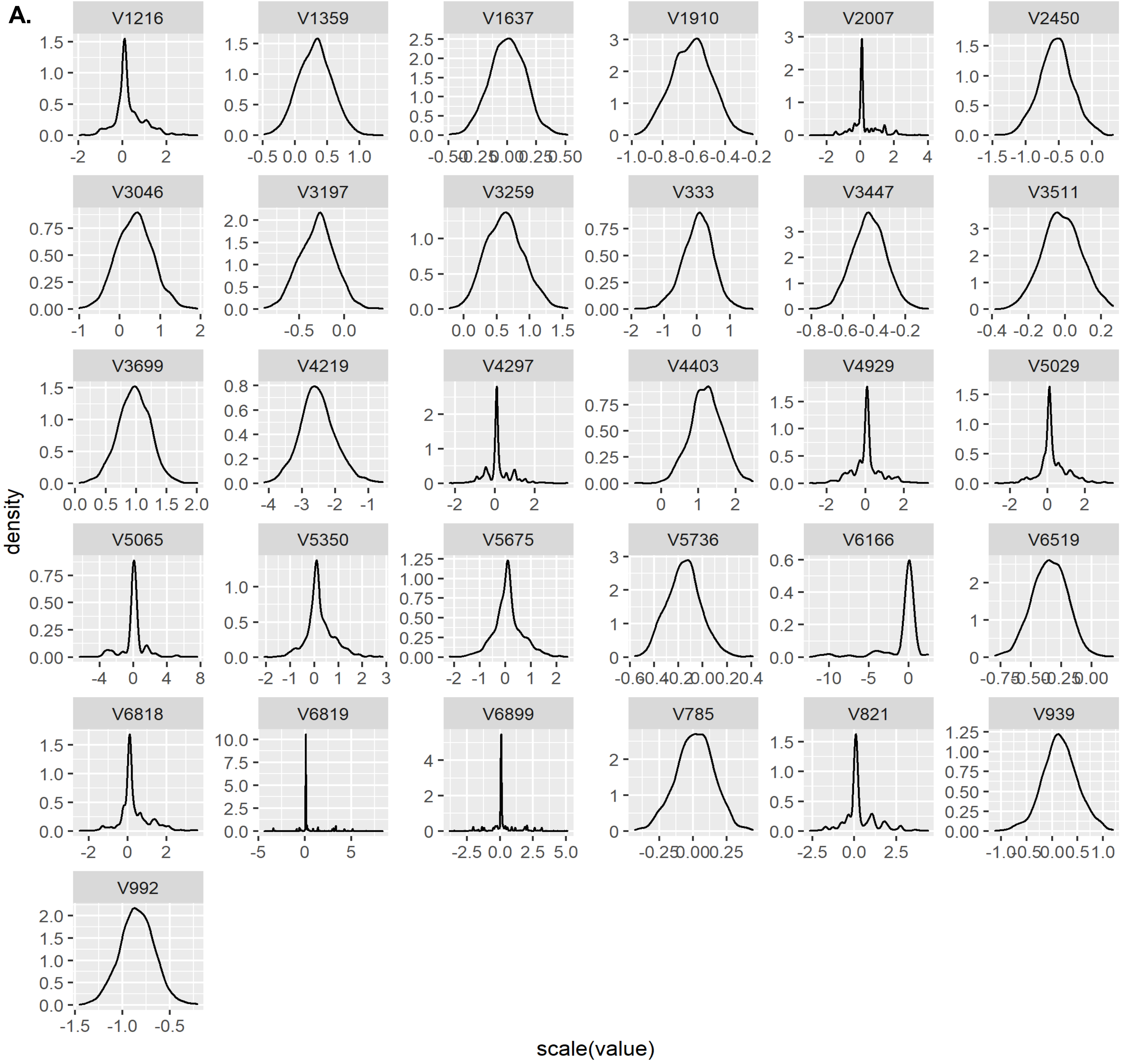

eFigure 2. The kernel density plot showing the distribution of each pathway-specific PRS.

31 PRS candidates with p value < 0.1 in association with 3-year mortality from univariate Cox regression model in the training dataset.

B.

Category	Pathway
AGE_tr	AGE_AT_ONSET(Binary)
V3259	GO_NEGATIVE_REGULATION_OF_ENDOTHELIAL_CELL_APOPTOTIC_PROCESS
V992	GO_HEMATOPOIETIC_STEM_CELL_DIFFERENTIATION
V1359	GO_NEGATIVE_REGULATION_OF_EPITHELIAL_CELL_APOPTOTIC_PROCESS
V6819	GO_POSITIVE_REGULATION_OF_AMYLOID_BETA_FORMATION
V2007	GO_REGULATION_OF_ODONTOGENESIS
V3447	GO_RESPONSE_TO_TUMOR_NECROSIS_FACTOR
V5675	GO_AMYLOID_BETA_METABOLIC_PROCESS
V4403	GO_PROTEASOMAL_UBIQUITIN_INDEPENDENT_PROTEIN_CATABOLIC_PROCESS
V821	GO_POSITIVE_REGULATION_OF_MEMBRANE_DEPOLARIZATION
V6519	GO_RECEPTOR_LOCALIZATION_TO_SYNAPSE
V2450	GO_GRANULOCYTE_DIFFERENTIATION
V1216	GO_REGULATION_OF_AMYLOID_PRECURSOR_PROTEIN_CATABOLIC_PROCESS
V4219	GO_SRP_DEPENDENT_COTRANSLATIONAL_PROTEIN_TARGETING_TO_MEMBRANE_TRANSLOCATION
V3699	GO_ZYMOGEN_ACTIVATION
V3046	GO_FIBRINOLYSIS
V5350	GO_AMYLOID_PRECURSOR_PROTEIN_CATABOLIC_PROCESS
V5736	GO_IMPORT_INTO_NUCLEUS
V6899	GO_POSITIVE_REGULATION_OF_AMYLOID_PRECURSOR_PROTEIN_CATABOLIC_PROCESS
V1637	GO_NEGATIVE_REGULATION_OF_HEMOPOIESIS
V4297	GO_SEROTONIN_RECEPTOR_SIGNALING_PATHWAY
V785	GO_ANTIGEN_PROCESSING_AND_PRESENTATION_OF_PEPTIDE_ANTIGEN
V6166	GO_MONOCYTE_AGGREGATION
V6818	GO_REGULATION_OF_AMYLOID_BETA_FORMATION
V333	GO_ENERGY_RESERVE_METABOLIC_PROCESS
V3197	GO_NEGATIVE_REGULATION_OF_MYELOID_CELL_DIFFERENTIATION
V1910	GO_RESPONSE_TO_INTERLEUKIN_1
V5065	GO_PROTEIN_LOCALIZATION_TO_PHAGOPHORE_ASSEMBLY_SITE
V5029	GO_AMYLOID_BETA_FORMATION
V3511	GO_T_CELL_DIFFERENTIATION
V939	GO_NEGATIVE_REGULATION_OF_MAPK_CASCADE
V4929	GO_GLOMERULAR_BASEMENT_MEMBRANE_DEVELOPMENT

eFigure 3.The standardized statistic of log-rank scores as a function of the hypothetical cutpoints of 31 candidate PRS identified by the univariate CoxPH model for the association with 3-year mortality.

The vertical dash line represents the cutpoint of PRS for the maximum of standardized log-rank statistic.

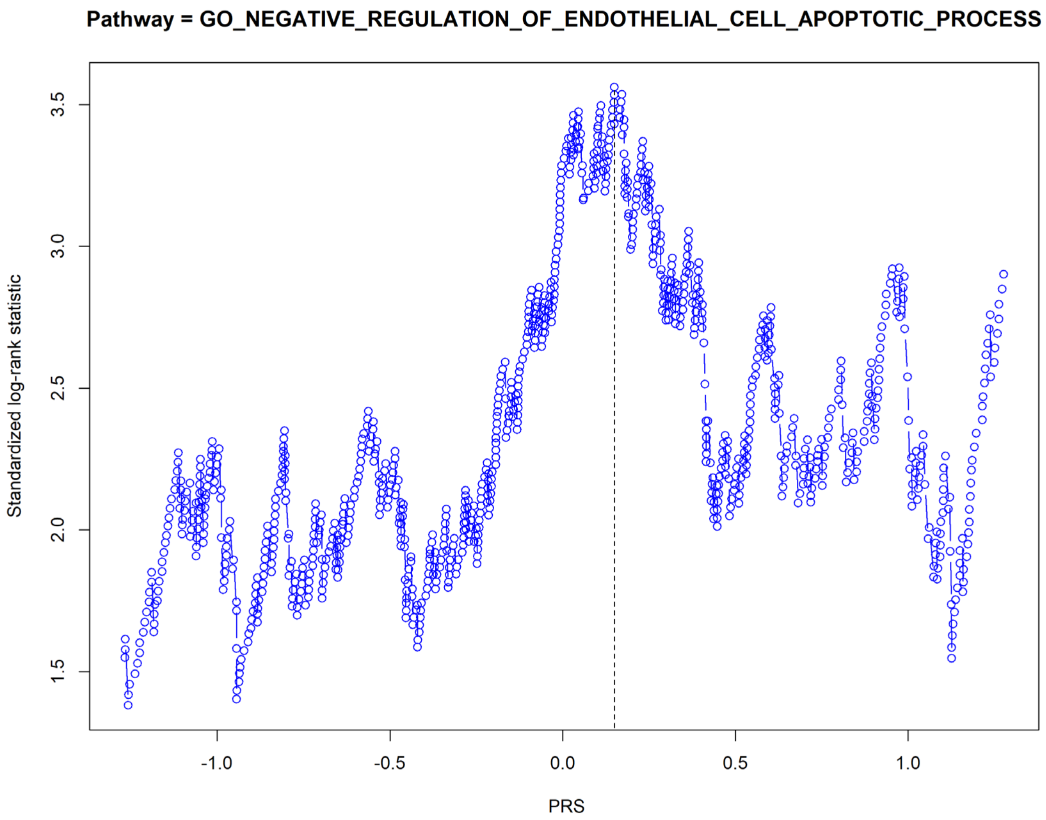

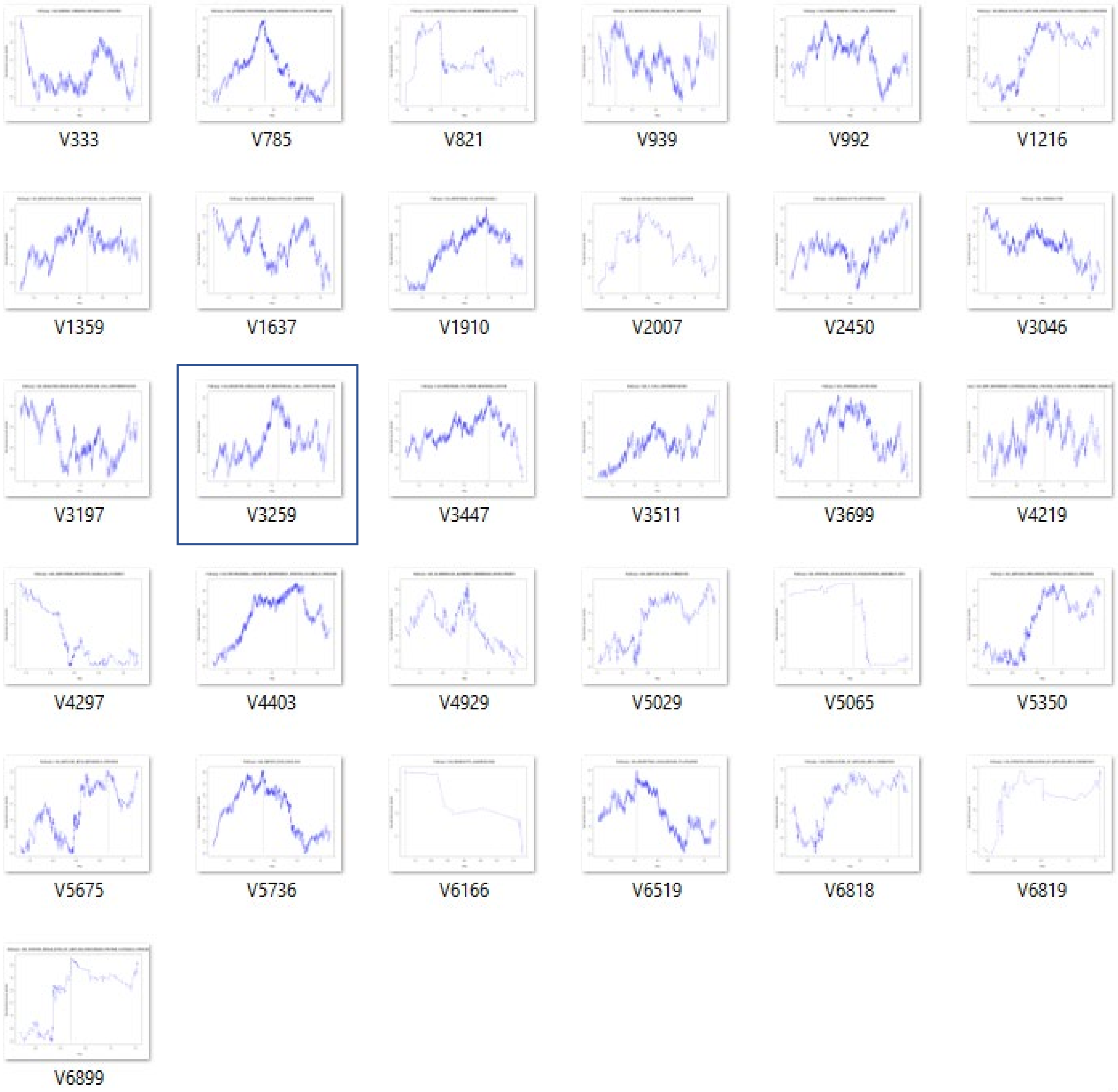

eFigure 4. Correlation matrix for the pathway-specific PRS, sex, and dichotomized age at index stroke.

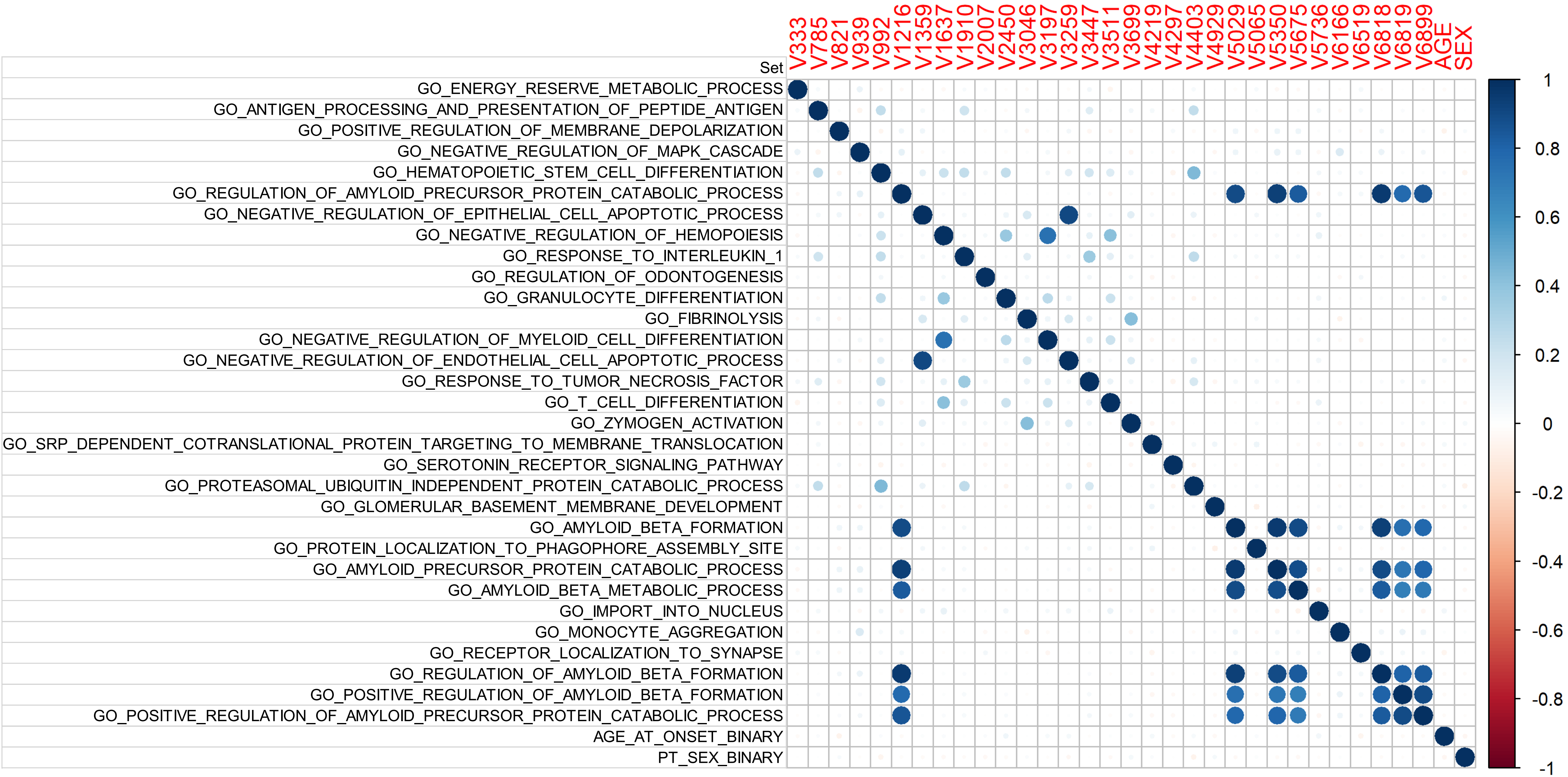

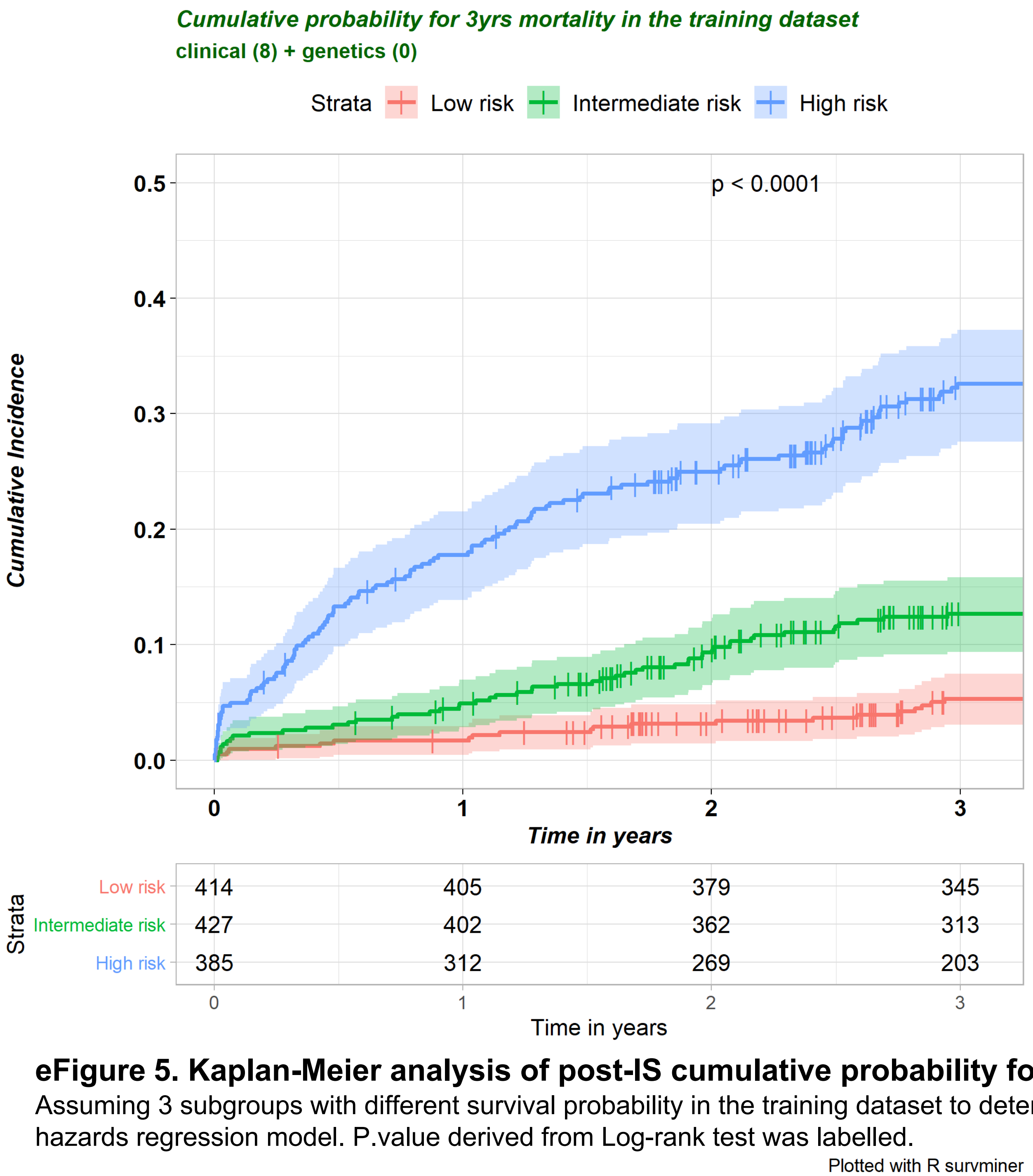

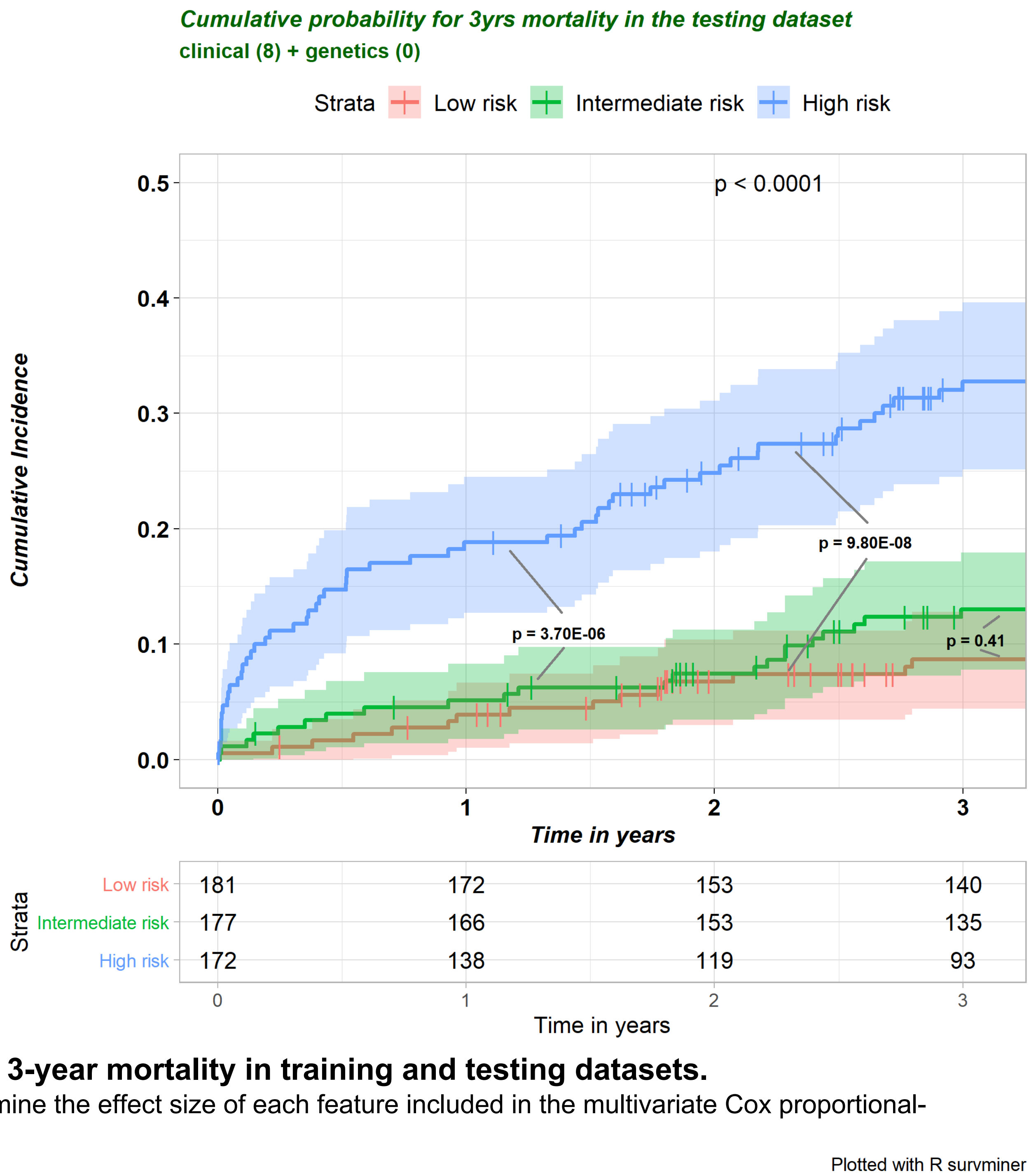

eFigure 5. Kaplan-Meier analysis of post-IS cumulative probability for 3-year mortality in training and testing datasets.
Assuming 3 subgroups with different survival probability in the training dataset to determine the effect size of each feature included in the multivariate Cox proportional-hazards regression model. P.value derived from Log-rank test was labelled.

eFigure 5.

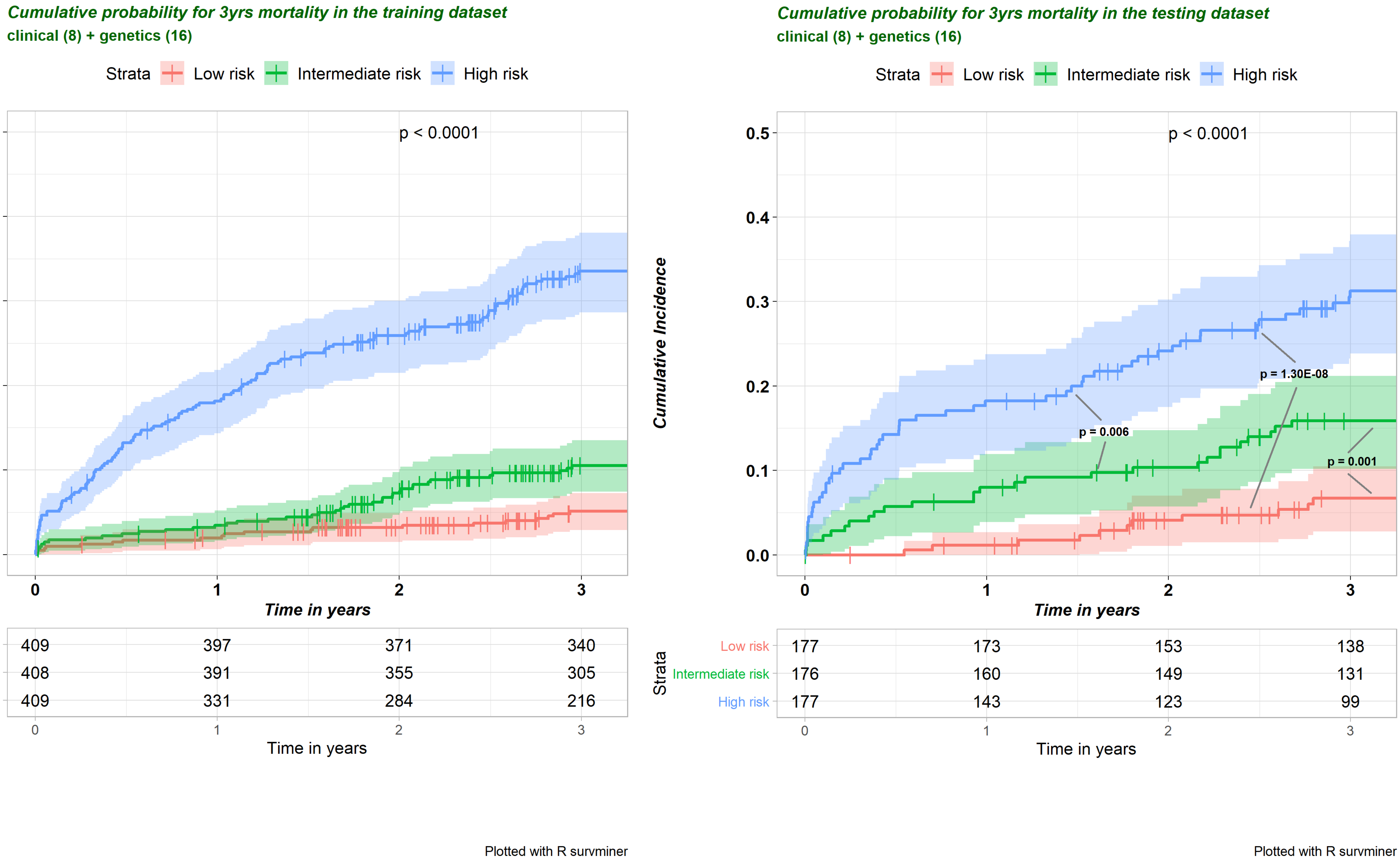
Cumulative probability for 3yrs mortality in the training dataset
clinical (8) + genetics (16)
Strata Low risk Intermediate risk High risk
p < 0.0001
Cumulative Incidence
0.5
0.4
0.3
0.2
0.1
0.0
0 1 2 3
Time in years
Strata
Low risk 409 397 371 340
Intermediate risk 408 391 355 305
High risk 409 331 284 216
0 1 2 3
Time in years
Plotted with R survminer
Cumulative probability for 3yrs mortality in the testing dataset
clinical (8) + genetics (16)
Strata Low risk Intermediate risk High risk
p < 0.0001
p = 1.30E-08
p = 0.006
p = 0.001
Cumulative Incidence
0.5
0.4
0.3
0.2
0.1
0.0
0 1 2 3
Time in years
Strata
Low risk 177 173 153 138
Intermediate risk 176 160 149 131
High risk 177 143 123 99
0 1 2 3
Time in years
Plotted with R survminer

eFigure 5. *Cumulative probability for 3yrs mortality in the training dataset*
clinical (8) + genetics (11)

Cumulative probability for 3yrs mortality in the testing dataset
clinical (8) + genetics (11)

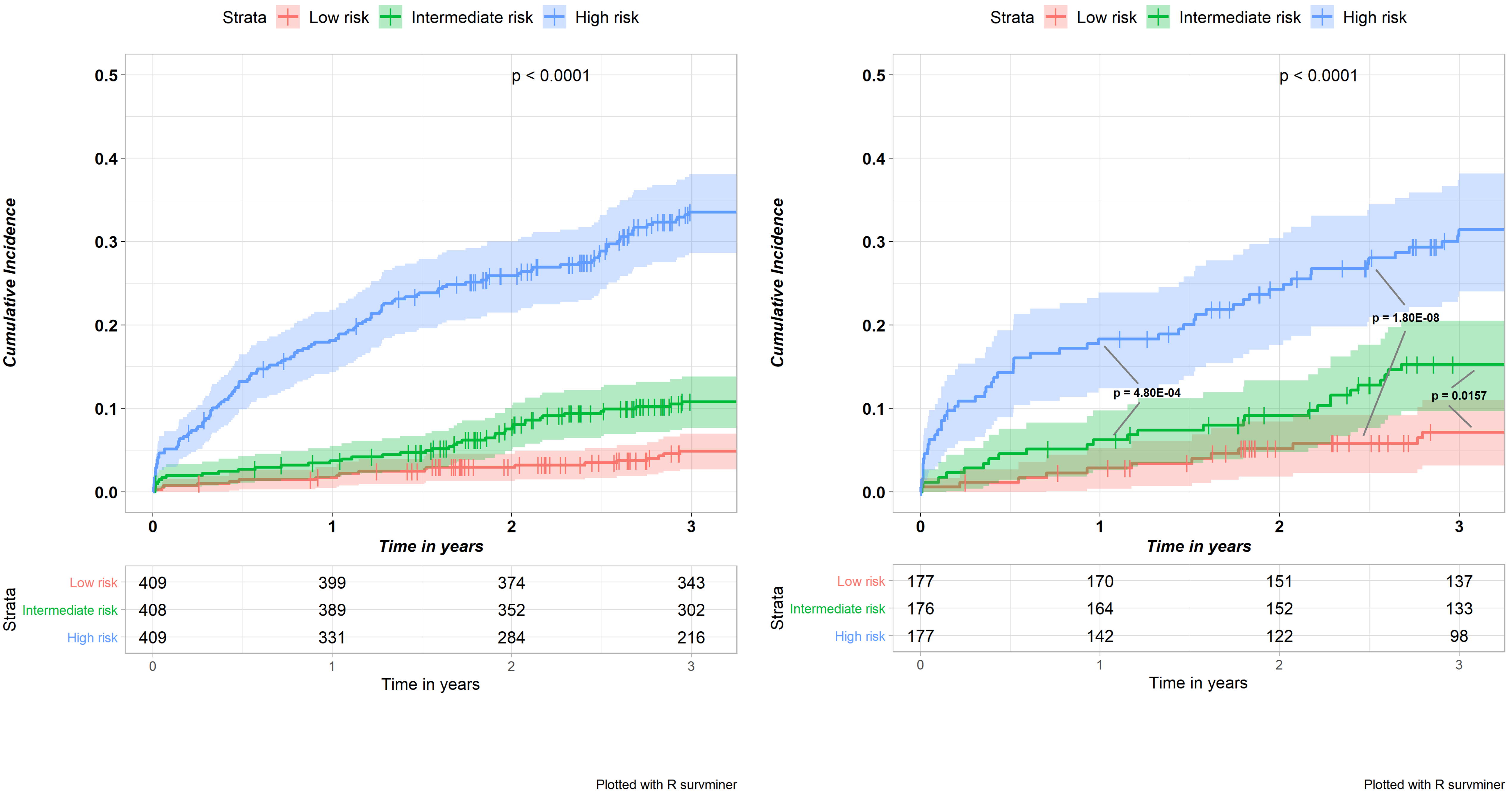

eFigure 5.

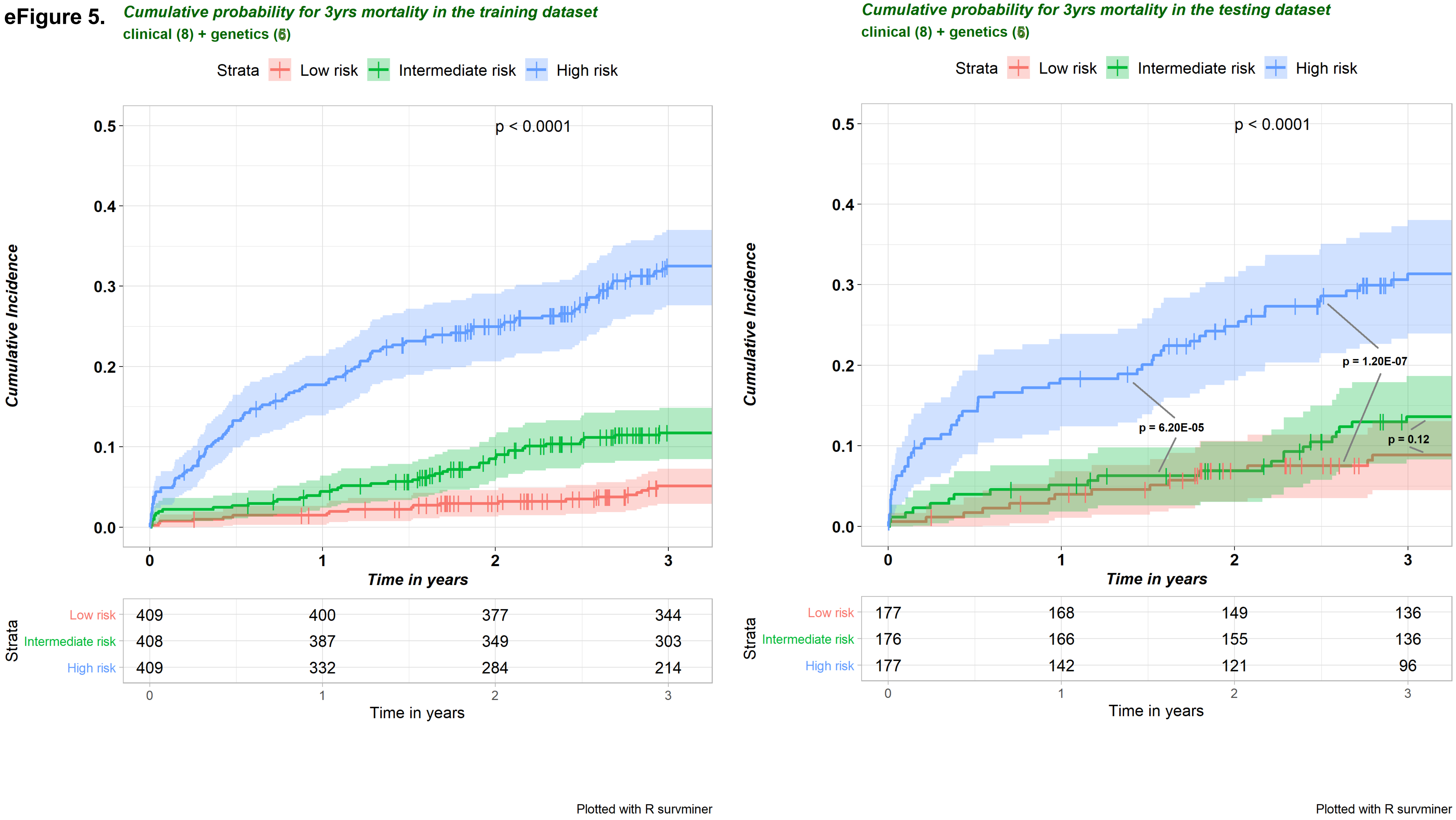
Cumulative probability for 3yrs mortality in the training dataset
clinical (8) + genetics (6)
Strata
Low risk
Intermediate risk
High risk
p < 0.0001
0.5
0.4
0.3
0.2
0.1
0.0
Cumulative Incidence
0
1
2
3
Time in years
Strata
Low risk 409 400 377 344
Intermediate risk 408 387 349 303
High risk 409 332 284 214
0
1
2
3
Time in years
Plotted with R survminer
Cumulative probability for 3yrs mortality in the testing dataset
clinical (8) + genetics (6)
Strata
Low risk
Intermediate risk
High risk
p < 0.0001
0.5
0.4
0.3
0.2
0.1
0.0
Cumulative Incidence
p = 1.20E-07
p = 6.20E-05
p = 0.12
0
1
2
3
Time in years
Strata
Low risk 177 168 149 136
Intermediate risk 176 166 155 136
High risk 177 142 121 96
0
1
2
3
Time in years
Plotted with R survminer

eFigure 5.

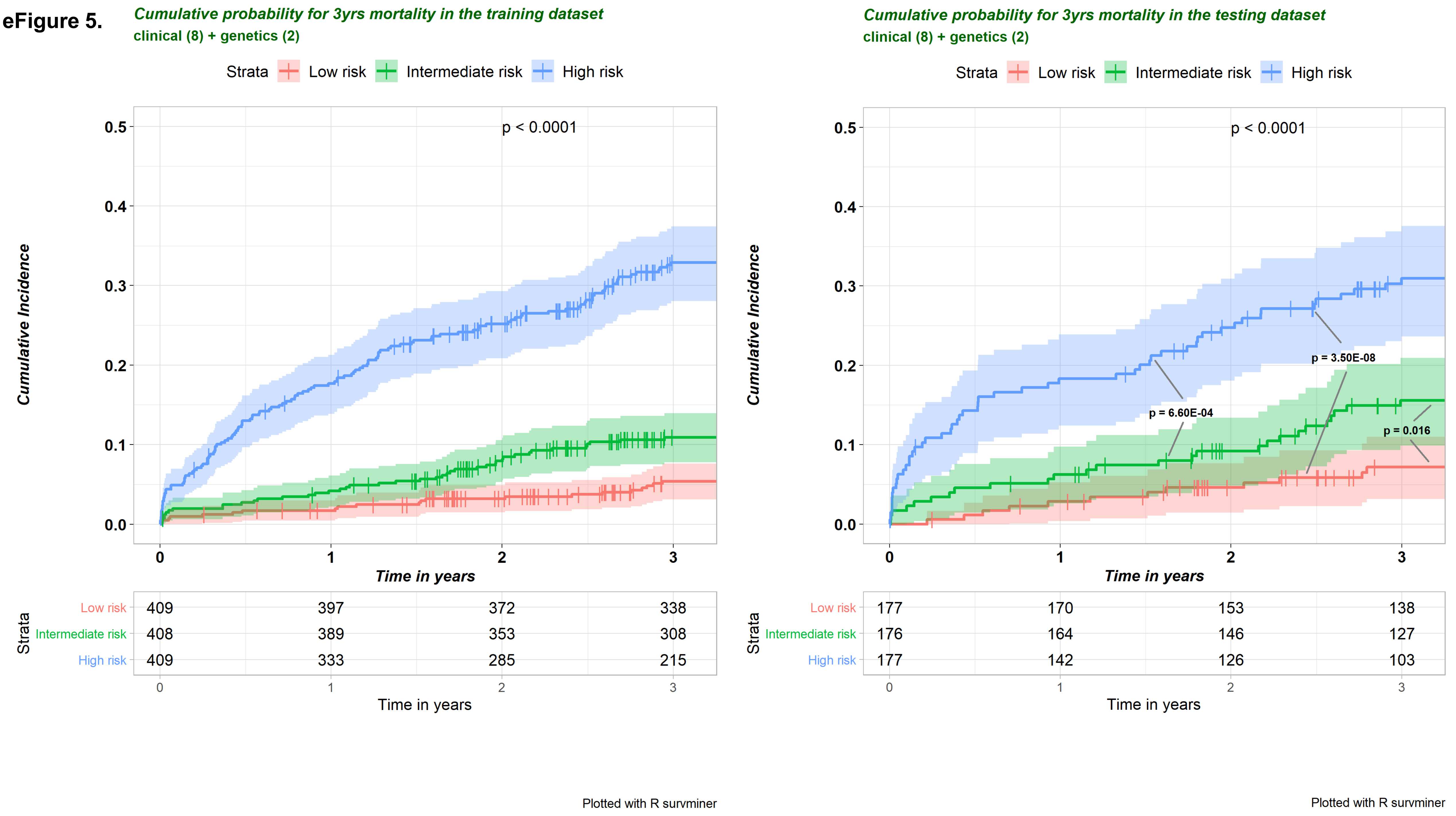

Cumulative probability for 3yrs mortality in the training dataset
clinical (8) + genetics (2)
Strata
Low risk
Intermediate risk
High risk
p < 0.0001
Cumulative Incidence
Time in years
Strata
Low risk 409 397 372 338
Intermediate risk 408 389 353 308
High risk 409 333 285 215
Time in years
Plotted with R survminer
Cumulative probability for 3yrs mortality in the testing dataset
clinical (8) + genetics (2)
Strata
Low risk
Intermediate risk
High risk
p < 0.0001
p = 3.50E-08
p = 6.60E-04
p = 0.016
Cumulative Incidence
Time in years
Strata
Low risk 177 170 153 138
Intermediate risk 176 164 146 127
High risk 177 142 126 103
Time in years
Plotted with R survminer

eFigure 6. The comparison of integrated and base models to determine the improvement of predictive models including additional features selected from pathway-specific PRSs.
A-D represents the two model comparisons between base and integrated models with additional 16, 11, 6, or 2 pathway-specific PRSs included, respectively. We calculated continuous NRI, IDI, and median improvement, as metrics to determine the improvement in prediction when comparing integrated model after additional features selected to the corresponding base model.

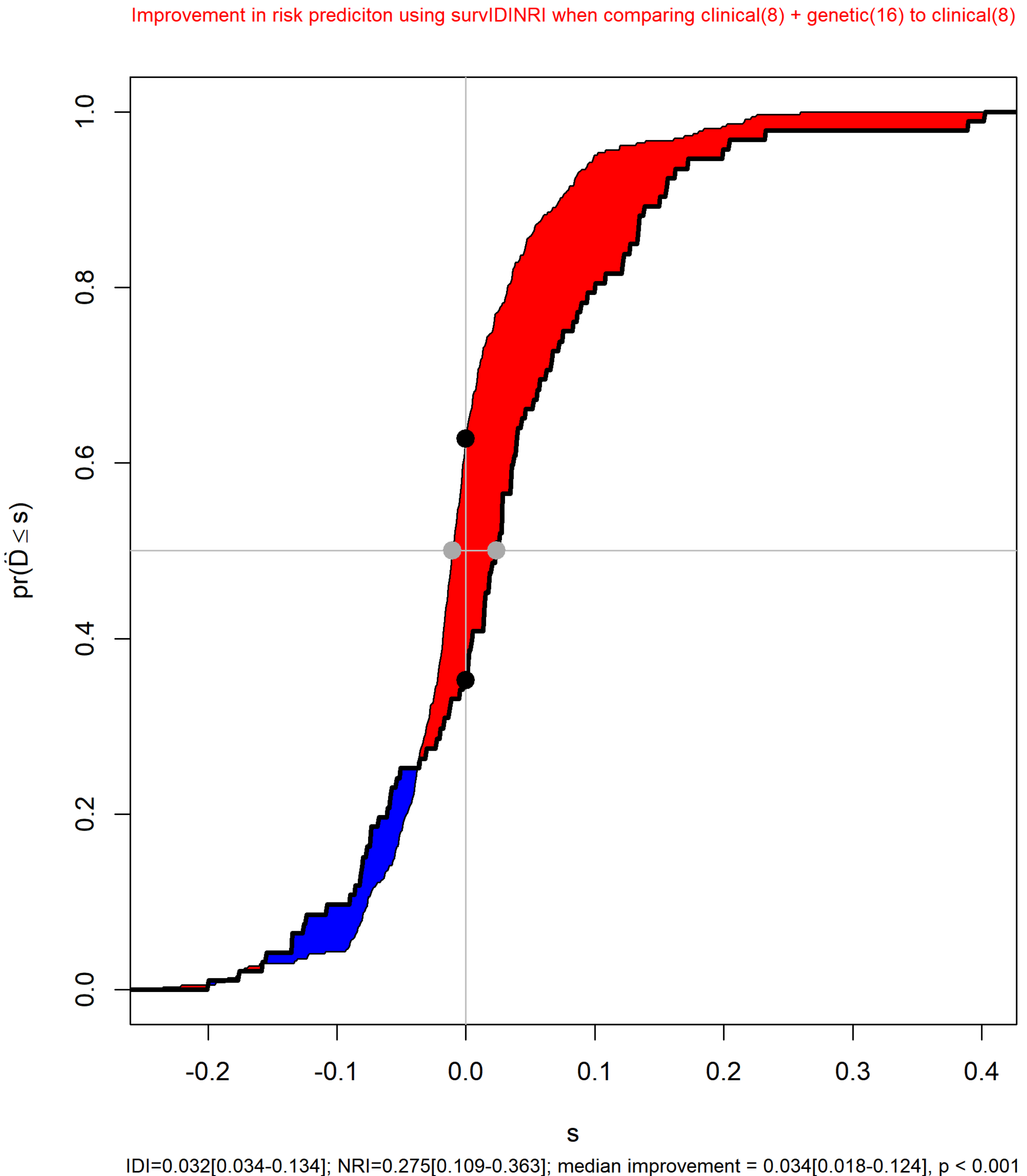

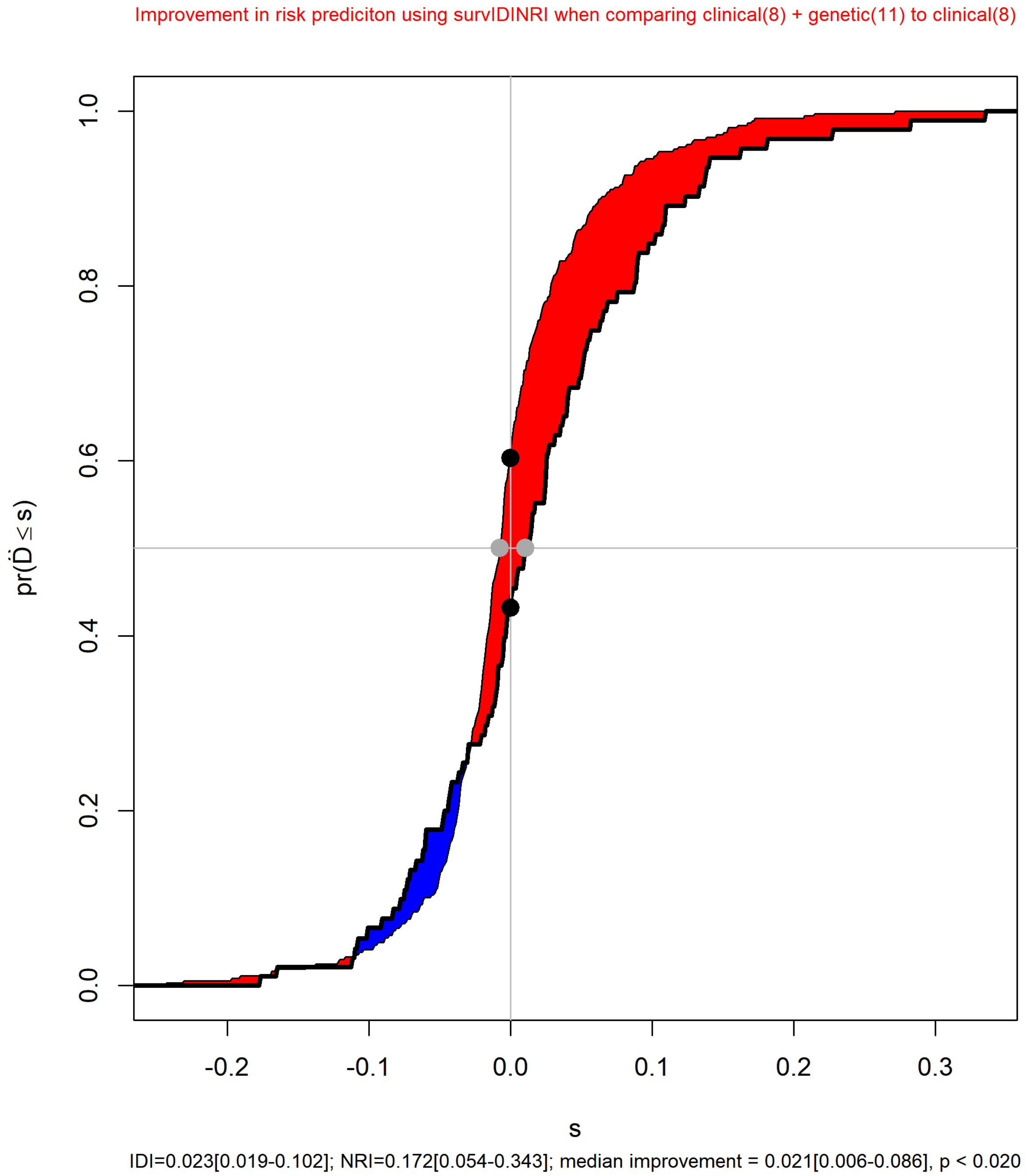

eFigure 6.

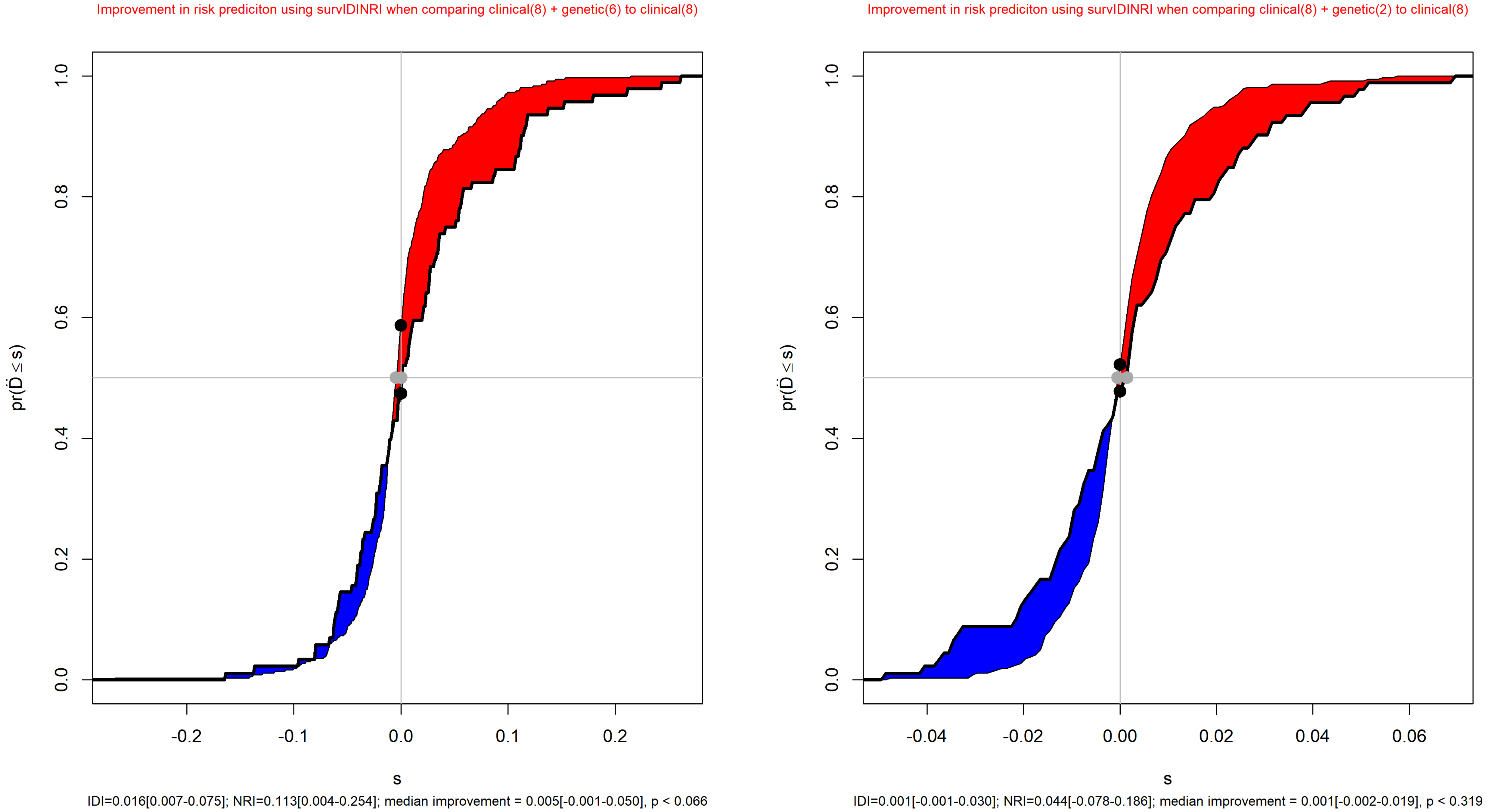

Improvement in risk prediciton using survIDINRI when comparing clinical(8) + genetic(6) to clinical(8)
pr(D̈ ≤ s)
s
IDI=0.016[0.007-0.075]; NRI=0.113[0.004-0.254]; median improvement = 0.005[-0.001-0.050], p < 0.066
Improvement in risk prediciton using survIDINRI when comparing clinical(8) + genetic(2) to clinical(8)
pr(D̈ ≤ s)
s
IDI=0.001[-0.001-0.030]; NRI=0.044[-0.078-0.186]; median improvement = 0.001[-0.002-0.019], p < 0.319

eFigure 7. Logistic regression to determine the association of each pathway-specific PRS with some clinical risk factors for ischemic stroke in all sample as well as subgroups stratified by the dichotomized age at index stroke.

A. older stroke subgroup; B. younger stroke subgroup.

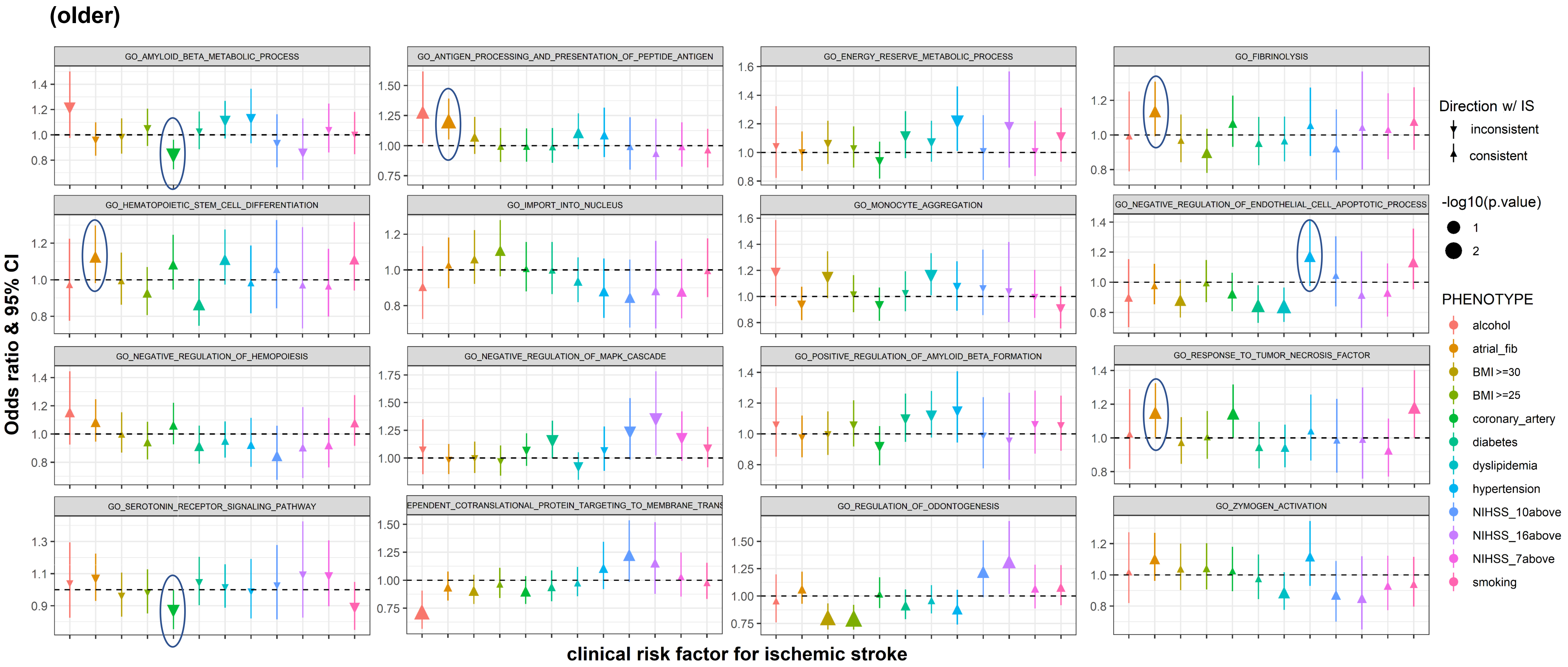

eFigure 7.

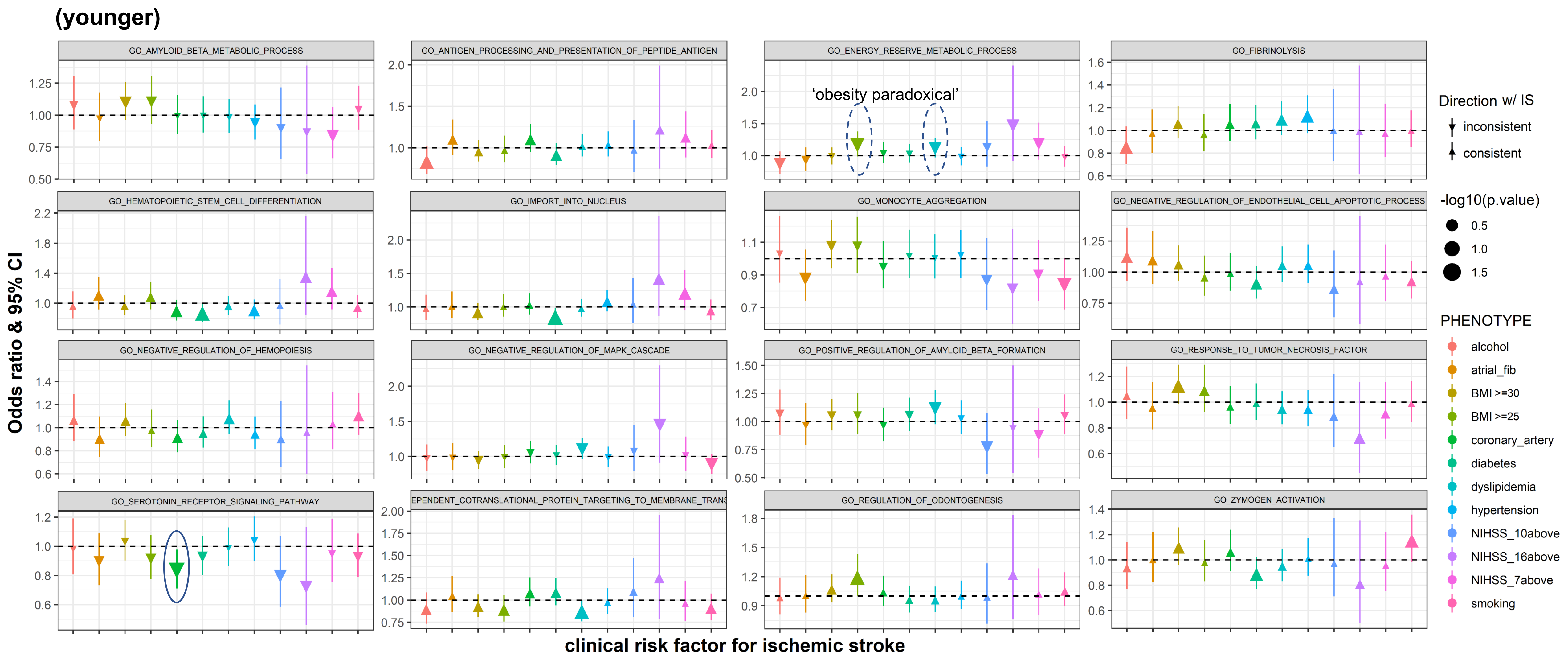
(younger)
GO_AMYLOID_BETA_METABOLIC_PROCESS
GO_ANTIGEN_PROCESSING_AND_PRESENTATION_OF_PEPTIDE_ANTIGEN
GO_ENERGY_RESERVE_METABOLIC_PROCESS
'obesity paradoxical'
GO_FIBRINOLYSIS
GO_HEMATOPOIETIC_STEM_CELL_DIFFERENTIATION
GO_IMPORT_INTO_NUCLEUS
GO_MONOCYTE_AGGREGATION
GO_NEGATIVE_REGULATION_OF_ENDOTHELIAL_CELL_APOPTOTIC_PROCESS
GO_NEGATIVE_REGULATION_OF_HEMOPOIESIS
GO_NEGATIVE_REGULATION_OF_MAPK_CASCADE
GO_POSITIVE_REGULATION_OF_AMYLOID_BETA_FORMATION
GO_RESPONSE_TO_TUMOR_NECROSIS_FACTOR
GO_SEROTONIN_RECEPTOR_SIGNALING_PATHWAY
EPENDENT_COTRANSLATIONAL_PROTEIN_TARGETING_TO_MEMBRANE_TRANS
GO_REGULATION_OF_ODONTOGENESIS
GO_ZYMOGEN_ACTIVATION
Odds ratio & 95% CI
clinical risk factor for ischemic stroke
Direction w/ IS
inconsistent
consistent
-log10(p.value)
0.5
1.0
1.5
PHENOTYPE
alcohol
atrial_fib
BMI >=30
BMI >=25
coronary_artery
diabetes
dyslipidemia
hypertension
NIHSS_10above
NIHSS_16above
NIHSS_7above
smoking
