# Supplementary material for: Predicting mortality among ischemic stroke patients using pathways-derived polygenic risk scores
Source: Sci Rep. 2022 Jul 19;12:12358. doi: 10.1038/s41598-022-16510-x (PMC9296485; doi:10.1038/s41598-022-16510-x)
Supplement: Supplementary file 2 — Supplementary Information 2. [file 41598_2022_16510_MOESM2_ESM.docx]

**Authors from Regeneron Genetics Center**

| Name | Location | Contribution |
| --- | --- | --- |
| Goncalo Abecasis, Ph.D. | RGC Management and Leadership Team, Regeneron Genetics Center, LLC, Tarrytown, NY | Securing funding, study design and oversight; Reviewing the final version of the manuscript. |
| Aris Baras, M.D. | RGC Management and Leadership Team, Regeneron Genetics Center, LLC, Tarrytown, NY | Securing funding, study design and oversight; Reviewing the final version of the manuscript. |
| Michael Cantor, M.D. | RGC Management and Leadership Team, Regeneron Genetics Center, LLC, Tarrytown, NY | Securing funding, study design and oversight; Reviewing the final version of the manuscript. |
| Giovanni Coppola, M.D. | RGC Management and Leadership Team, Regeneron Genetics Center, LLC, Tarrytown, NY | Securing funding, study design and oversight; Reviewing the final version of the manuscript. |
| Aris Economides, Ph.D. | RGC Management and Leadership Team, Regeneron Genetics Center, LLC, Tarrytown, NY | Securing funding, study design and oversight; Reviewing the final version of the manuscript. |
| Luca A. Lotta, M.D., Ph.D. | RGC Management and Leadership Team, Regeneron Genetics Center, LLC, Tarrytown, NY | Securing funding, study design and oversight; Reviewing the final version of the manuscript. |
| John D. Overton, Ph.D. | RGC Management and Leadership Team, Regeneron Genetics Center, LLC, Tarrytown, NY | Securing funding, study design and oversight; Reviewing the final version of the manuscript. |
| Jeffrey G. Reid, Ph.D. | RGC Management and Leadership Team, Regeneron Genetics Center, LLC, Tarrytown, NY | Securing funding, study design and oversight; Reviewing the final version of the manuscript. |
| Alan Shuldiner, M.D. | RGC Management and Leadership Team, Regeneron Genetics Center, LLC, Tarrytown, NY | Securing funding, study design and oversight; Reviewing the final version of the manuscript. |
| Christina Beechert | Sequencing and Lab Operations, Regeneron Genetics Center, LLC, Tarrytown, NY | performed and are responsible for sample genotyping; performed and are responsible for exome sequencing |
| Caitlin Forsythe, M.S. | Sequencing and Lab Operations, Regeneron Genetics Center, LLC, Tarrytown, NY | performed and are responsible for sample genotyping; performed and are responsible for exome sequencing |
| Erin D. Fuller | Sequencing and Lab Operations, Regeneron Genetics Center, LLC, Tarrytown, NY | performed and are responsible for exome sequencing |
| Zhenhua Gu, M.S. | Sequencing and Lab Operations, Regeneron Genetics Center, LLC, Tarrytown, NY | conceived and are responsible for laboratory automation |
| Michael Lattari | Sequencing and Lab Operations, Regeneron Genetics Center, LLC, Tarrytown, NY | performed and are responsible for exome sequencing |
| Alexander Lopez, M.S. | Sequencing and Lab Operations, Regeneron Genetics Center, LLC, Tarrytown, NY | performed and are responsible for sample genotyping; performed and are responsible for exome sequencing; conceived and are responsible for laboratory automation |
| John D. Overton, Ph.D. | Sequencing and Lab Operations, Regeneron Genetics Center, LLC, Tarrytown, NY | performed and are responsible for sample genotyping; performed and are responsible for exome sequencing; conceived and are responsible for laboratory automation; sample tracking and the library information management system |
| Thomas D. Schleicher, M.S. | Sequencing and Lab Operations, Regeneron Genetics Center, LLC, Tarrytown, NY | conceived and are responsible for laboratory automation |
| Maria Sotiropoulos Padilla, M.S. | Sequencing and Lab Operations, Regeneron Genetics Center, LLC, Tarrytown, NY | performed and are responsible for exome sequencing |
| Karina Toledo | Sequencing and Lab Operations, Regeneron Genetics Center, LLC, Tarrytown, NY | Performed and are responsible for sample genotyping; performed and are responsible for exome sequencing |
| Louis Widom | Sequencing and Lab Operations, Regeneron Genetics Center, LLC, Tarrytown, NY | performed and are responsible for exome sequencing |
| Sarah E. Wolf, M.S. | Sequencing and Lab Operations, Regeneron Genetics Center, LLC, Tarrytown, NY | performed and are responsible for exome sequencing |
| Manasi Pradhan, M.S. | Sequencing and Lab Operations, Regeneron Genetics Center, LLC, Tarrytown, NY | sample tracking and the library information management system |
| Kia Manoochehri | Sequencing and Lab Operations, Regeneron Genetics Center, LLC, Tarrytown, NY | sample tracking and the library information management system |
| Ricardo H. Ulloa | Sequencing and Lab Operations, Regeneron Genetics Center, LLC, Tarrytown, NY | sample tracking and the library information management system |
| Xiaodong Bai, Ph.D. | Genome Informatics, Regeneron Genetics Center, LLC, Tarrytown, NY | performed and are responsible for analysis needed to produce exome and genotype data |
| Suganthi Balasubramanian, Ph.D. | Genome Informatics, Regeneron Genetics Center, LLC, Tarrytown, NY | provide variant and gene annotations and their functional interpretation of variants |
| Leland Barnard, Ph.D. | Genome Informatics, Regeneron Genetics Center, LLC, Tarrytown, NY | conceived and are responsible for creating, developing, and deploying analysis platforms and computational methods for analyzing genomic data |
| Andrew Blumenfeld | Genome Informatics, Regeneron Genetics Center, LLC, Tarrytown, NY | conceived and are responsible for creating, developing, and deploying analysis platforms and computational methods for analyzing genomic data |
| Gisu Eom | Genome Informatics, Regeneron Genetics Center, LLC, Tarrytown, NY | provided compute infrastructure development and operational support |
| Lukas Habegger, Ph.D. | Genome Informatics, Regeneron Genetics Center, LLC, Tarrytown, NY | conceived and are responsible for creating, developing, and deploying analysis platforms and computational methods for analyzing genomic data |
| Alicia Hawes, B.S. | Genome Informatics, Regeneron Genetics Center, LLC, Tarrytown, NY | performed and are responsible for analysis needed to produce exome and genotype data |
| Shareef Khalid | Genome Informatics, Regeneron Genetics Center, LLC, Tarrytown, NY | provide variant and gene annotations and their functional interpretation of variants |
| Jeffrey G. Reid, Ph.D. | Genome Informatics, Regeneron Genetics Center, LLC, Tarrytown, NY | performed and are responsible for analysis needed to produce exome and genotype data; provided compute infrastructure development and operational support; provide variant and gene annotations and their functional interpretation of variants; conceived and are responsible for creating, developing, and deploying analysis platforms and computational methods for analyzing genomic data |
| Evan K. Maxwell, Ph.D. | Genome Informatics, Regeneron Genetics Center, LLC, Tarrytown, NY | conceived and are responsible for creating, developing, and deploying analysis platforms and computational methods for analyzing genomic data |
| William Salerno, Ph.D. | Genome Informatics, Regeneron Genetics Center, LLC, Tarrytown, NY | performed and are responsible for analysis needed to produce exome and genotype data |
| Jeffrey C. Staples, Ph.D. | Genome Informatics, Regeneron Genetics Center, LLC, Tarrytown, NY | conceived and are responsible for creating, developing, and deploying analysis platforms and computational methods for analyzing genomic data |
| Marcus B. Jones, Ph.D. | Research Program Management, Regeneron Genetics Center, LLC, Tarrytown, NY | contributed to the management and coordination of all research activities, planning and execution; contributed to the review process for the final version of the manuscript |
| Lyndon J. Mitnaul, Ph.D. | Research Program Management, Regeneron Genetics Center, LLC, Tarrytown, NY | contributed to the management and coordination of all research activities, planning and execution; contributed to the review process for the final version of the manuscript |
